# Sex and Age Dependencies of Aqueductal Cerebrospinal Fluid Dynamics Parameters in Healthy Subjects

**DOI:** 10.3389/fnagi.2019.00199

**Published:** 2019-08-02

**Authors:** Thomas Sartoretti, Michael Wyss, Elisabeth Sartoretti, Carolin Reischauer, Nicolin Hainc, Nicole Graf, Christoph Binkert, Arash Najafi, Sabine Sartoretti-Schefer

**Affiliations:** ^1^Laboratory of Translational Nutrition Biology, Department of Health Sciences and Technology, ETH Zürich, Zurich, Switzerland; ^2^Philips Healthcare, Zurich, Switzerland; ^3^Department of Radiology, Cantonal Hospital Winterthur, Winterthur, Switzerland; ^4^Department of Medicine, University of Fribourg, Fribourg, Switzerland; ^5^Department of Radiology, HFR Fribourg – Hôpital Cantonal, Fribourg, Switzerland; ^6^Department of Neuroradiology, University Hospital Zürich, University of Zürich, Zurich, Switzerland; ^7^Graf Biostatistics, Winterthur, Switzerland

**Keywords:** magnetic resonance imaging, cerebrospinal fluid, phase contrast MRI, flow dynamics, aqueduct, sex, age

## Abstract

**Objectives:**

To assess the influence of age and sex on 10 cerebrospinal fluid (CSF) flow dynamics parameters measured with an MR phase contrast (PC) sequence within the cerebral aqueduct at the level of the intercollicular sulcus.

**Materials and Methods:**

128 healthy subjects (66 female subjects with a mean age of 52.9 years and 62 male subjects with a mean age of 51.8 years) with a normal Evans index, normal medial temporal atrophy (MTA) score, and without known disorders of the CSF circulation were included in the study. A PC MR sequence on a 3T MR scanner was used. Ten different flow parameters were analyzed using postprocessing software. Ordinal and linear regression models were calculated.

**Results:**

The parameters stroke volume (sex: *p* < 0.001, age: *p* = 0.003), forward flow volume (sex: *p* < 0.001, age: *p* = 0.002), backward flow volume (sex: *p* < 0.001, age: *p* = 0.018), absolute stroke volume (sex: *p* < 0.001, age: *p* = 0.005), mean flux (sex: *p* < 0.001, age: *p* = 0.001), peak velocity (sex: *p* = 0.009, age: *p* = 0.0016), and peak pressure gradient (sex: *p* = 0.029, age: *p* = 0.028) are significantly influenced by sex and age. The parameters regurgitant fraction, stroke distance, and mean velocity are not significantly influenced by sex and age.

**Conclusion:**

CSF flow dynamics parameters measured in the cerebral aqueduct are partly age and sex dependent. For establishment of reliable reference values for clinical use in future studies, the impact of sex and age should be considered and incorporated.

## Introduction

Cerebrospinal fluid is a water-like transparent fluid produced mainly in the choroid plexus in the ventricles of the brain but also in the interstitium and the meninges. CSF acts as a cushion for the brain and is secreted and absorbed continuously. The overall CSF turnover ranges from three to five times per day whereby 90–150 ml of CSF resides within the CSF space at a given time. After exiting the lateral ventricles through the interventricular foramina of Monro, CSF travels to the third ventricle before passing through the cerebral aqueduct into the fourth ventricle. Finally, CSF reaches subarachnoid spaces at the craniocervical junction via the Foramen of Magendie and Foramina of Luschka. From here, CSF can either flow to the villous sites of absorption over the cerebral hemispheres or to the spinal subarachnoid space (Sakka et al., 2011; Tumani et al., 2017). When passing through the cerebral aqueduct CSF flow is not static but rather oscillatory depending on breathing and heart beat (Markenroth Bloch et al., 2018). An established (Luetmer et al., 2002; Lee et al., 2004; Schmid Daners et al., 2012; Najafi et al., 2018) and reliable (Balédent et al., 2004; Tain and Alperin, 2009; Najafi et al., 2018) method to quantify CSF flow in the cerebral aqueduct is PC MRI in MR scanners of varying field strengths (Lee et al., 2004; Battal et al., 2011). Commercially available postprocessing software then calculates specific parameters (Lee et al., 2004; Battal et al., 2011; Najafi et al., 2018). Certain parameters can deviate strongly from normal values in some pathologies such as in normal pressure hydrocephalus (NPH), aqueduct stenosis, or Chiari malformation (Bradley et al., 1996; Luetmer et al., 2002; Stoquart-El Sankari et al., 2009; Gulbiz Kartal and Algin, 2014; Bradley, 2015; Yamada et al., 2015; Markenroth Bloch et al., 2018). Thus CSF flow values are of interest in a clinical setting. Interestingly, in a previous study, age and sex (yet not height) have been described to also significantly impact different CSF flow dynamics parameters in the aqueduct (Schmid Daners et al., 2012). However, only two parameters (stroke volume and average flow) were analyzed with a limited sample size of subjects. Furthermore, only two age categories with subjects aged 24 years versus 70 years on average were included in the study. Therefore, in our study we aimed at reinvestigating sex and age dependencies of 10 CSF flow dynamics parameters in 128 healthy subjects of varying age (ranging from 17 to 88 years) within the cerebral aqueduct using a PC MR sequence (Wåhlin et al., 2012; Najafi et al., 2018).

## Materials and Methods

This study was carried out in accordance with the recommendations of the Cantonal Ethical Committee Zurich, Zurich, Switzerland with written informed consent from all subjects. All subjects gave written informed consent in accordance with the Declaration of Helsinki. This prospective study with the number BASEC 2017-00129 was approved by the Cantonal Ethical Committee Zurich, Zurich, Switzerland on 12 April 2017. Signed informed consent was obtained from all subjects.

### Subject Selection

Between June 2016 and July 2018 the images and data of 135 neurologically healthy subjects aged between 17 and 88 years were collected. Subjects were considered healthy if the ventricles were not enlarged based on an Evans Index below 0.28 in subjects aged 50 years or younger and below 0.31 in subjects older than 50 years (Brix et al., 2017), if no aqueduct stenosis was diagnosed on anatomical 3D T_2_w TSE DRIVE images and if no disorder of the CSF circulation was known. Additionally subjects had to present with a normal medial temporal atrophy (MTA) score on precontrast T_1_w images with a score of 0 or 1 in subjects up to the age of 75 years and with a score of 0, 1, or 2 in subjects older than 75 years (Scheltens et al., 1992; Barkhof et al., 2007). Thus a neurodegenerative disease (Alzheimer’s disease) as well as mild cognitive impairment could be excluded, because a strong relationship between MTA scores and Alzheimer pathology (as well as cognitive impairment) is known (Scheltens et al., 1992; Barkhof et al., 2007). Furthermore, subjects had to present without any cardiovascular diseases, such as cardiac arrhythmia and arterial hypertension (if present and known) had to be controlled by administration of drugs. Seven subjects had to be excluded.

Sixty-six female (mean age of 52.9 years, *SD* of 20.24 years) and 62 male subjects (mean age of 51.8 years, *SD* of 19.59 years) without significant age difference (*p* = 0.75) thus totaling 128 subjects were finally included in the study.

### MRI Imaging

All examinations were performed with an eight channel head coil on a 3Tesla Achieva scanner (Philips Healthcare, Best, Netherlands). A sagittal 3D T_1_w TFE sequence with a slice thickness of 1 mm was used both for anatomical and morphological evaluation of the hippocampus and for planning of the additional MR sequences.

An MR PC sequence optimized specifically in terms of reliability and reproducibility for CSF flow quantification in the cerebral aqueduct was utilized, as recommended in a previous study (Najafi et al., 2018). The imaging parameters of the PC sequence are depicted in Table 1. In this previous study, the accuracy of the MR PC sequence for low flow rates and the impact of sequence parameters on the accuracy of measurements were studied by pumping physiological saline solutions through flexible tubes with MR injectors followed by MR imaging. The effect of VENC, resolution, and slice thickness on the accuracy of flow rate measurements was assessed and optimal parameter values were determined. Additionally, optimal ROI and slice placement was checked (Najafi et al., 2018).

**TABLE 1:** Sequence parameters of the PC MR sequence used.

**Parameters**	**PC MR sequence**
Field of view (FoV)	150 × 150 mm^2^
Maximal acquired voxel size	0.5 × 0.5 × 3.0 mm^3^
Reconstructed voxel size	0.45 × 0.45 × 3.0 mm^3^
Number of slices	1
Repetition time (TR)	13 ms
Echo time (TE)	8.0 ms
Flip angle	15°
Number of signal averages (NSA)	1
Receiver bandwidth	216 Hz/pixel
Velocity encoding (VENC)	22 cm/s
Cardiac synchronization	Peripheral pulse triggering
Heart phases	12–16
Acquisition time in min	4–7 min (heart rate dependent)

A retrospective cardiac gating via peripheral pulse device, known as peripheral pulse triggering, was performed. Subjects with cardiac arrhythmia were excluded from the study. LPC together with background noise filtering was used to correct for phase offsets due to eddy currents as well for offsets due to concomitant gradients. LPC is implemented by the MR vendor and is applied during reconstruction of the PC sequence. LPC eliminates the need for manual background phase offset correction. For precise anatomical depiction of the aqueduct, a 3D T_2_w TSE DRIVE sequence in sagittal orientation (sequence parameters depicted in the Supplementary Material) was utilized.

**FIGURE 1:**
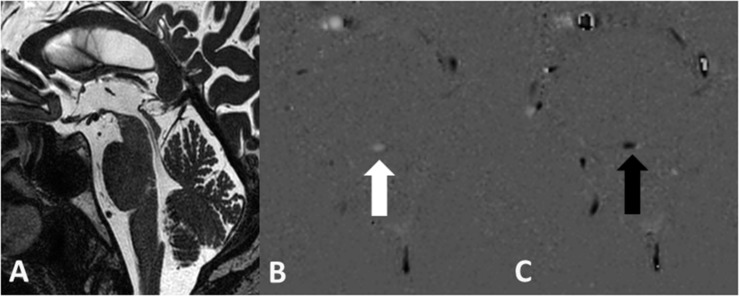
Normal aqueduct with even width in a 70-year-old male subject on the sagittal 3D T_2_w TSE DRIVE sequence **(A)**. Grayscale encoded flow direction with hyperintense caudocranial **(B)** and hypointense craniocaudal **(C)** flow direction.

A normal aqueduct presented with an even width without focal narrowing or presence of intraluminal septa or webs (Figure 1A). The single transversal slice of the PC sequence was always planned at the level of the intercollicular sulcus on the midline sagittal 3D T_2_w TSE DRIVE sequence.

### Postprocessing

The PC sequence generates magnitude and phase images; in our case, an anatomical magnitude image, a magnitude PC image, and the phase of the PC image (PCA/P). A processing software on an independent workstation IntelliSpace Portal version 8 (Philips Healthcare, Best, Netherlands) then calculates the flow parameters from the PCA/P image with the help of a program called “MR Q flow analysis” (Onen et al., 2005; Salm et al., 2007). Apart from having to manually define a ROI around the cerebral aqueduct, the software operates fully automatically. The ROI was always placed in a manner that ensured that only a minimum amount of tissue surrounding the aqueduct was included within its borders; however, the minimum size of the ROI had to be 10 pixels according to the software requirements (Najafi et al., 2018). The postprocessing was done within 1 min. Different flow parameters were obtained from the postprocessing software: “Forward flow volume” (ml), “backward flow volume” (ml), “regurgitation fraction” (%), “absolute stroke volume” (ml), “mean flux” (ml/s), “stroke distance” (cm), “mean velocity” (cm/s), “peak velocity” (cm/s), and “peak pressure gradient (mmHg)” (Najafi et al., 2018). The software calculates values up to two decimal places. Detailed information on these flow parameters is given in Table 2.

**TABLE 2:** Detailed information on the flow parameters obtained from post-processing software.

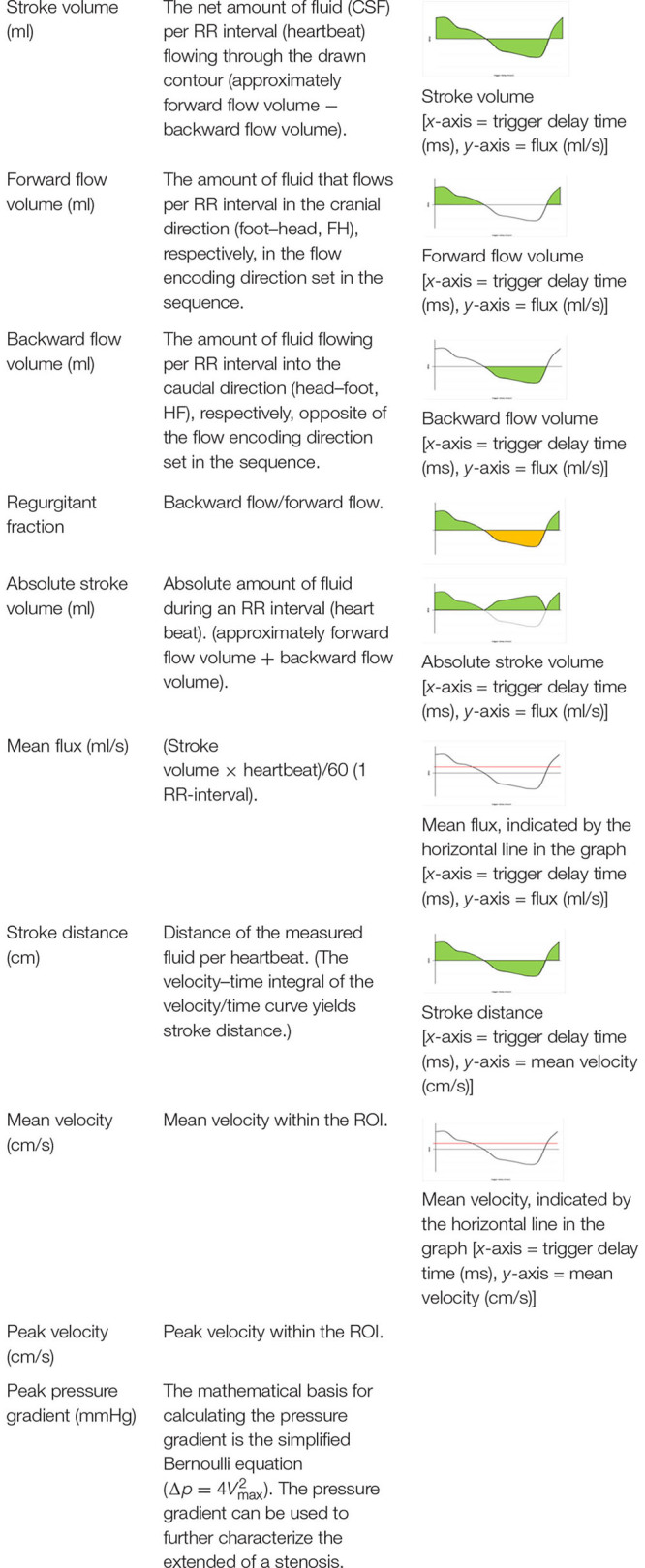

Flow velocity and the flow direction are vendor-dependent encoded and presented in the PC phase grayscale image. The setting of the direction of the VENC parameter in the MR sequence defines the appearance of the signal in the PCA/P image. In a transversal acquisition plane the encoding of the flow direction is feet–head. During the diastole, CSF flows in the VENC direction from feet to head, meaning forward flow, yielding a bright signal. During the systole, CSF flows in the opposite direction head to feet, corresponding to backward flow, yielding a dark signal. Thus, hyperintense signal in the PCA/P image signifies caudo-cranial flow and hypointense signal in the PCA/P image signifies cranio-caudal flow on the “MR Q flow analysis tool” (Figures 1B,C). The setting of the VENC in the PC sequence is crucial. A VENC of 22 cm/s was chosen as recommended by a previous study (Najafi et al., 2018). The VENC should be the same as or only slightly different from the expected flow velocity in order to obtain higher signal in the phase image and to prevent phase aliasing (Battal et al., 2011; Yamada et al., 2015).

### Statistical Analysis

The 10 CSF flow dynamics parameters were analyzed with linear or ordinal regression models with predictors age and sex. For linear models, linearity was checked with component and residual plots. Homoscedasticity was checked with residual versus fitted values plot and tested with the standardized Breusch–Pagan test. Normality was checked with a Q–Q plot of the standardized residuals. For ordinal models, the proportional odds assumption was checked with a test of nominal effects and with a graphic by stratifying on each predictor and computing the logits of all proportions of the form *Y* ≥ *j*, *j* = 1,2, …, *k*. The level of significance was set at *p* < 0.05.

All analyses were performed in the R programming language (version 3.3.3) (R Core Team, 2017). The package “MASS” (Venables and Ripley, 2002) was used to compute the ordinal regression models. The package “ggplot2” was used to visualize the data by fitting the various endpoints by age, stratified for men and women. All data are available as the Supplementary Material.

## Results

**FIGURE 2:**
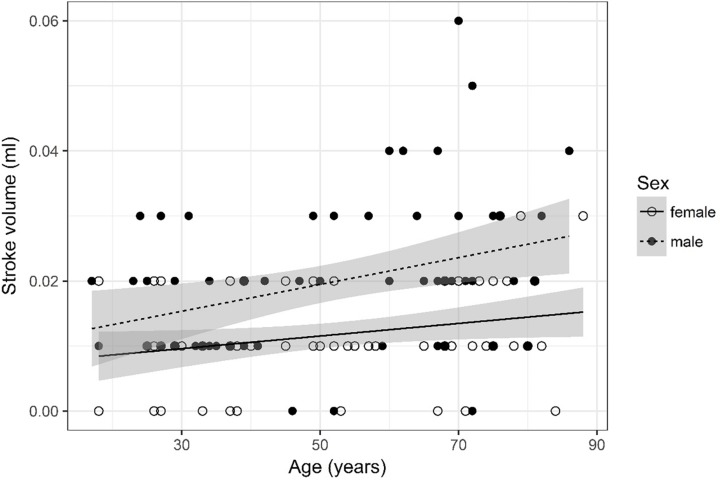
Scatter plot visualizing stroke volume. A regression line for the formula stroke volume ∼ age for both men and women was added with corresponding 95% CIs (gray zones).

**FIGURE 3:**
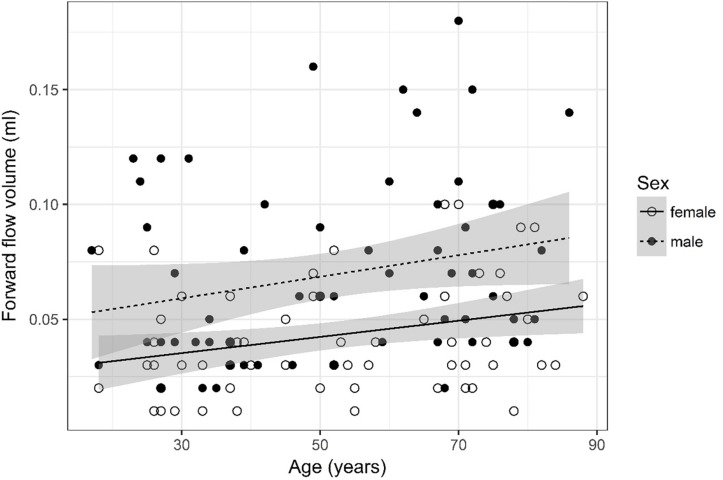
Scatter plot visualizing forward flow volume. A regression line for the formula forward flow volume ∼ age for both men and women was added with corresponding 95% CIs (gray zones).

**FIGURE 4:**
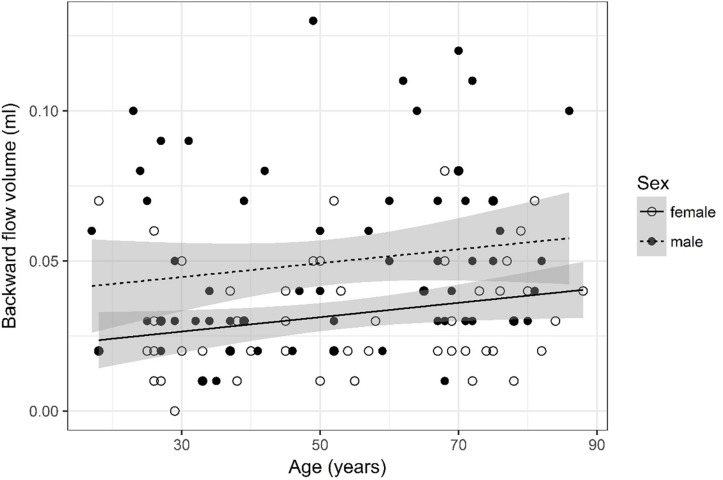
Scatter plot visualizing backward flow volume. A regression line for the formula backward flow volume ∼ age for both men and women was added with corresponding 95% CIs (gray zones).

**FIGURE 5:**
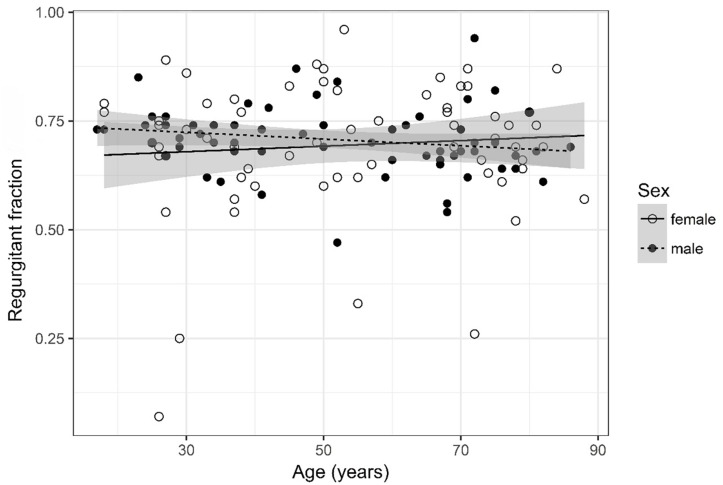
Scatter plot visualizing regurgitant fraction. A regression line for the formula regurgitant fraction ∼ age for both men and women was added with corresponding 95% CIs (gray zones).

**FIGURE 6:**
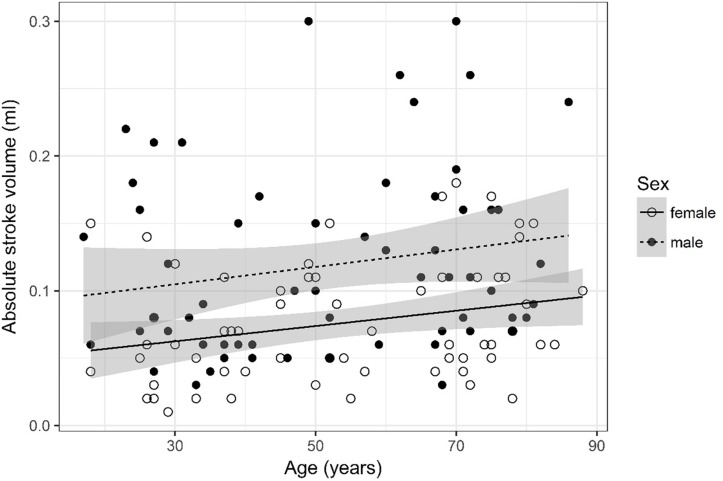
Scatter plot visualizing absolute stroke volume. A regression line for the formula absolute stroke volume ∼ age for both men and women was added with corresponding 95% CIs (gray zones).

**FIGURE 7:**
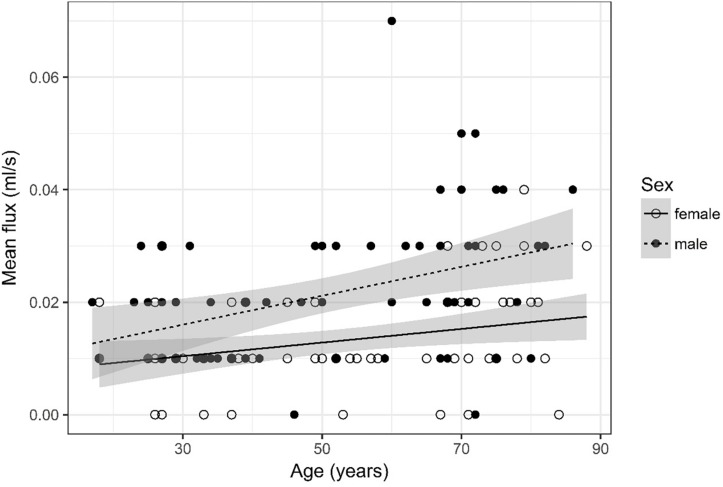
Scatter plot visualizing mean flux. A regression line for the formula mean flux ∼ age for both men and women was added with corresponding 95% CIs (gray zones).

**FIGURE 8:**
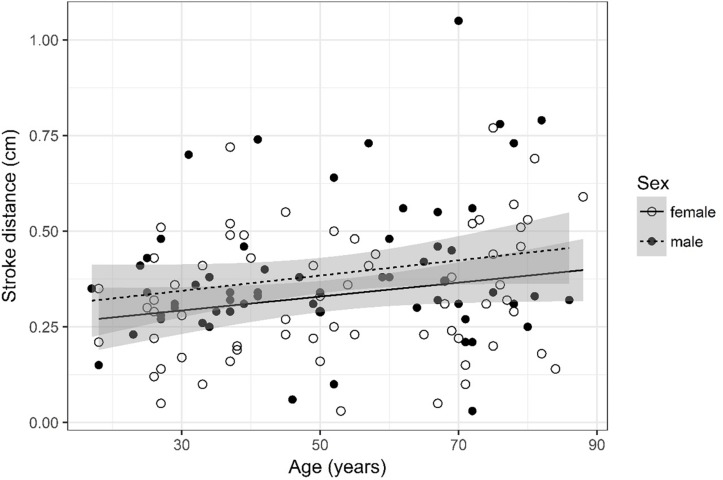
Scatter plot visualizing stroke distance. A regression line for the formula stroke distance ∼ age for both men and women was added with corresponding 95% CIs (gray zones).

**FIGURE 9:**
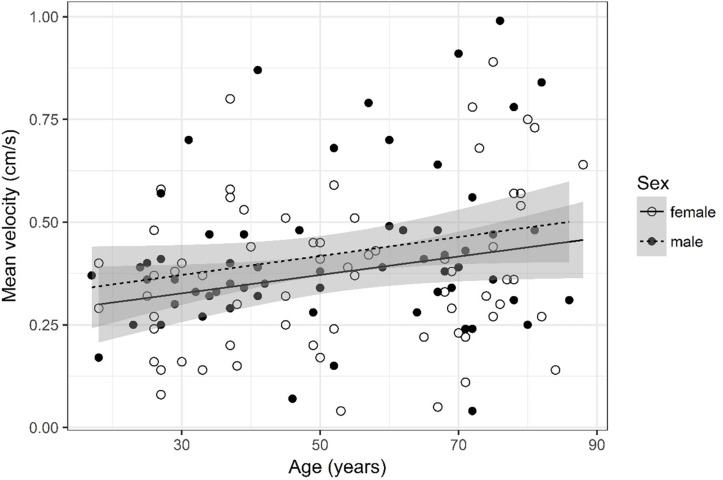
Scatter plot visualizing mean velocity. A regression line for the formula mean velocity ∼ age for both men and women was added with corresponding 95% CIs (gray zones).

**FIGURE 10:**
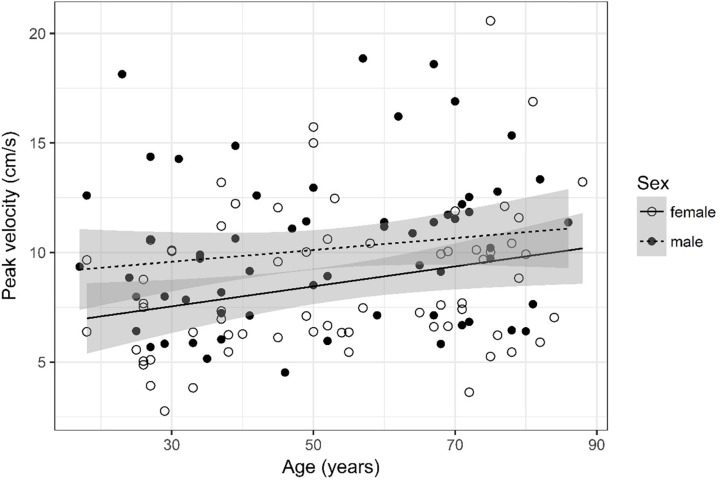
Scatter plot visualizing peak velocity. A regression line for the formula peak velocity ∼ age for both men and women was added with corresponding 95% CIs (gray zones).

**FIGURE 11:**
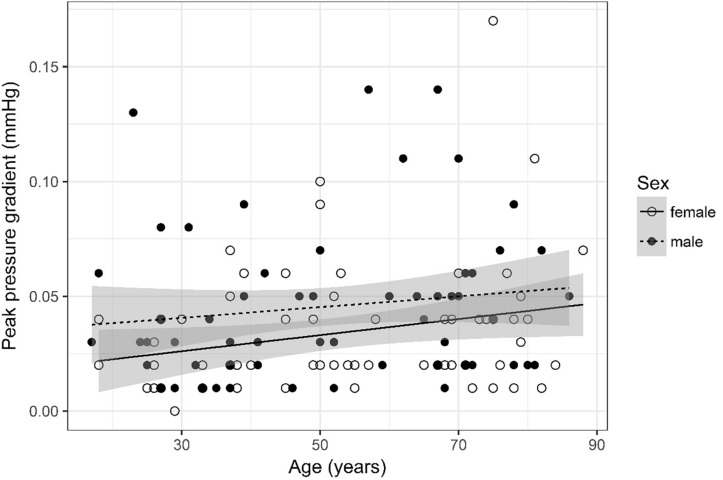
Scatter plot visualizing peak pressure gradient. A regression line for the formula peak pressure gradient ∼ age for both men and women was added with corresponding 95% CIs (gray zones).

A detailed overview of the data is given in Table 3. As most parameters are significantly impacted by age and sex the data are depicted separately for each sex and for two age (17–50 and 51–88) categories [based on the same considerations as used in the subject selection to determine normal ventricular size (Brix et al., 2017)] using the mean, SD, and 95% CIs. For visual purposes scatter plots (Figures 2–11) depict the influence of age and sex on CSF flow dynamics parameters. As expected, neither age (*p* = 0.934) nor sex (*p* = 0.232) influenced the heart rate (measured in BPM) of subjects that was measured as part of retrospective cardiac gating. A scatter plot depicting the data can be found in the Supplementary Material.

**TABLE 3:** Depiction of data of all 10 CSF flow dynamics parameters separated by sex and age using the mean, SD, and 95% CIs.

	**Male (17–50) (*n* = 31)**	**Male (51–88) (*n* = 31)**	**Female (17–50) (*n* = 32)**	**Female (51–88) (*n* = 34)**
**Flow parameter**	**mean; SD; 95% CI**	**mean; SD; 95% CI**	**mean; SD; 95% CI**	**mean; SD; 95% CI**
Stroke volume (ml)	0.015; 0.008; [0.013; 0.018]	0.024; 0.014; [0.019; 0.029]	0.011; 0.007; [0.008; 0.013]	0.013; 0.009; [0.010; 0.016]
Forward flow volume (ml)	0.060; 0.037; [0.046; 0.073]	0.079; 0.041; [0.065; 0.093]	0.038; 0.021; [0.031; 0.045]	0.048; 0.028; [0.039; 0.058]
Backward flow volume (ml)	0.045; 0.030; [0.035; 0.056]	0.054; 0.030; [0.044; 0.065]	0.028; 0.017; [0.022; 0.034]	0.036; 0.021; [0.029; 0.043]
Regurgitant fraction	0.726; 0.062; [0.704; 0.748]	0.688; 0.090; [0.656; 0.720]	0.685; 0.170; [0.626; 0.744]	0.702; 0.140; [0.655; 0.750]
Absolute stroke volume (ml)	0.106; 0.067; [0.082; 0.129]	0.132; 0.070; [0.107; 0.157]	0.067; 0.038; [0.054; 0.081]	0.083; 0.047; [0.067; 0.099]
Mean flux (ml/s)	0.016; 0.008; [0.013; 0.019]	0.027; 0.015; [0.022; 0.032]	0.012; 0.007; [0.009; 0.014]	0.015; 0.010; [0.011; 0.018]
Stroke distance (cm)	0.345; 0.130; [0.299; 0.391]	0.430; 0.219; [0.353; 0.508]	0.310; 0.154; [0.257; 0.363]	0.358; 0.179; [0.298; 0.418]
Mean velocity (cm/s)	0.374; 0.145; [0.322; 0.425]	0.470; 0.227; [0.390; 0.549]	0.347; 0.166; [0.289; 0.404]	0.407; 0.211; [0.336; 0.478]
Peak velocity (cm/s)	9.496; 3.312; [8.330; 10.662]	10.822; 3.641; [9.540; 12.104]	8.095; 3.245; [6.970; 9.219]	9.050; 3.459; [7.887; 10.212]
Peak pressure gradient (mmHg)	0.040; 0.028; [0.030; 0.050]	0.052; 0.035; [0.039; 0.064]	0.031; 0.024; [0.022; 0.039]	0.037; 0.032; [0.027; 0.048]

### Stroke Volume (Figure 2)

Values were recoded into four categories, i.e., 0, 0.01, 0.02, and =0.03. Furthermore, four age categories for stroke volume were defined (17–34, 35–52, 53–71, and 72–88) for assessment of odds.

The ordinal regression indicates that both sex (*p* < 0.001) and age (*p* = 0.003) are significant predictors for stroke volume. As age increases by one category, the odds of observing category 4 of stroke volume versus the other three categories increase by a factor of 1.03 [95% CI (1; 1.05)]. The odds of observing category 4 of stroke volume versus the other three categories are 4.59 times higher [95% CI (2.31; 9.4)] for male than for female subjects.

### Forward Flow Volume (Figure 3)

Forward flow volume data was logarithmized. The analysis of variance table indicates that both sex (*p* < 0.001) and age (*p* = 0.002) are significant predictors for forward flow volume. Values are higher for older compared to younger subjects and male compared to female subjects. The linear model [*F*(2,125) = 15.83, *p* < 0.001, adjusted *R*^2^ = 0.18] reveals that male sex leads to a 21.5% increase in forward flow volume [95% CI (12.5%; 30.5%)] compared to female sex. An increase of 1 year in age leads to a 0.36% increase in forward flow volume [95% CI (0.14%; 0.59%)].

### Backward Flow Volume (Figure 4)

Backward flow volume was logarithmized. The analysis of variance table indicates that both sex (*p* < 0.001) and age (*p* = 0.018) are significant predictors for backward flow volume. Values were higher for older compared to younger subjects and male compared to female subjects. The linear model [*F*(2,124) = 10.33, *p* < 0.001, adjusted *R*^2^ = 0.12] reveals that male sex leads to a 18.5% increase in backward flow volume [95% CI (9.24%; 27.77%)] compared to female sex. An increase of 1 year in age leads to a 0.28% increase in backward flow volume [95% CI (0.05%; 0.52%)].

### Regurgitant Fraction (Figure 5)

Regurgitant fraction was logarithmized. The analysis of variance table indicates that neither sex (*p* = 0.502) nor age (*p* = 0.559) are significant predictors for regurgitant fraction. The interaction between age and sex was also tested but did not prove to be significant.

### Absolute Stroke Volume (Figure 6)

Absolute stroke volume was logarithmized. The analysis of variance table indicates that both sex (*p* < 0.001) and age (*p* = 0.005) are significant predictors for absolute stroke volume. Values were higher for older compared to younger subjects and male compared to female subjects. The linear model [*F*(2,125) = 14.12, *p* < 0.001, adjusted *R*^2^ = 0.17] reveals that male sex leads to a 21.1% increase in absolute stroke volume [95% CI (12%; 30.38%)] compared to female sex. An increase of 1 year in age leads to a 0.33% increase in absolute stroke volume [95% CI (0.1%; 0.57%)].

### Mean Flux (Figure 7)

Mean flux was recoded into four categories, i.e., 0, 0.01, 0.02, and =0.03. Furthermore, four age categories for mean flux were defined (17–34, 35–52, 53–71, and 72–88) for assessment of odds.

The ordinal regression indicates that both sex (*p* < 0.001) and age (*p* < 0.001) are significant predictors for mean flux. As age increases by one category, the odds of observing category 4 of mean flux versus the other three categories increase by a factor of 1.03 [95% CI (1.01; 1.05)]. The odds of observing category 4 of mean flux versus the other three categories are 4.51 times higher [95% CI (2.27; 9.24)] for male than for female subjects.

### Stroke Distance (Figure 8)

Stroke distance was logarithmized. The analysis of variance table indicates that neither sex (*p* = 0.083) nor age (*p* = 0.114) are significant predictors for stroke distance.

### Mean Velocity (Figure 9)

Mean velocity was logarithmized. The analysis of variance table indicates that neither sex (*p* = 0.141) nor age (*p* = 0.084) are significant predictors for mean velocity.

### Peak Velocity (Figure 10)

The analysis of variance table indicates that both sex (*p* = 0.009) and age (*p* = 0.016) are significant predictors for peak velocity. The linear model [*F*(2,124) = 6.49, *p* = 0.002, adjusted *R*^2^ = 0.08] reveals that male sex leads to a 1.62 cm/s increase in peak velocity [95% CI (0.43; 2.8)] compared to female sex. An increase of 1 year in age leads to a 0.037 cm/s increase in peak velocity [95% CI (0.007; 0.07)].

### Peak Pressure Gradient (Figure 11)

The analysis of variance table indicates that both sex (*p* = 0.029) and age (*p* = 0.028) are significant predictors for peak pressure gradient. The linear model [*F*(2,124) = 4.89, *p* = 0.009, adjusted *R*^2^ = 0.06] reveals that male sex leads to a 0.012 mmHg increase in peak pressure gradient [95% CI (0.001; 0.02)] compared to female sex. An increase of 1 year in age leads to a 0.0003 mmHg increase in peak pressure gradient [95% CI (3 ^*^ 10^–5^; 6 ^*^ 10^–4^)].

## Discussion

Quantitative PC MR imaging in the cerebral aqueduct is a reliable and established method to assess CSF flow dynamics (Enzmann and Pelc, 1993; Barkhof et al., 1994; Balédent et al., 2004; Tain and Alperin, 2009). We utilized a PC MR sequence that was specifically designed for reliable and reproducible quantification of CSF flow dynamics in the aqueduct (Najafi et al., 2018). An accurate interpretation of the flow parameters obtained after postprocessing of the PC sequence is possible if the flow parameters are also correlated with anatomical information obtained on the high resolution anatomical 3D T_2_w DRIVE sequence. For example, high peak velocity is observed in NPH and in aqueductal stenosis, but in NPH the aqueduct is wide and in aqueductal stenosis septa formation in the aqueduct is demonstrated on the 3D T_2_w DRIVE sequence. Therefore, the 3D T_2_w DRIVE sequence plays a decisive role as stenosis or septa formation within the cerebral aqueduct are only reliably seen on this sequence. In healthy patients the aqueduct shows no irregularities or stenoses, thus the flow parameters within a homogenously formed aqueduct should not differ.

Comparison of CSF flow values from different studies may, however, be misleading (Bradley et al., 1996; Bradley, 2015; Yamada et al., 2015). Flow values are sometimes calculated differently depending on the postprocessing method chosen. For example in a recent paper (Bradley et al., 1996), the stroke volume was averaged over the diastolic and systolic fluxes. In the evaluation software we used, the absolute stroke volume is given as the sum of systolic and diastolic flow. To compare the two values, the absolute stroke volume should be divided by two. In our study we used an established postprocessing software called “MR Q flow analysis” that has been validated in several studies (Onen et al., 2005; Salm et al., 2007).

Concerning age and sex dependence a previous study has revealed that the age and sex of subjects influence CSF flow values in the aqueduct. Stroke volume and average flow within the aqueduct were analyzed. While significantly higher aqueductal CSF stroke volumes and average flow rates for males compared to females were found, neither age nor height significantly impacted these parameters (Schmid Daners et al., 2012).

In our study, we demonstrate both sex and age dependence in select flow parameters. All flow parameters analyzed in this study are significantly impacted by sex and age except for regurgitant fraction, stroke distance, and mean velocity; and values increase with age and in the case of male sex. For example, in the parameters forward flow volume and absolute stroke volume, male sex leads to an increase of 21.5 and 21.1% in values while an increase of 1 year in age leads to a 0.36 and 0.33% increase in values. Thus, the effect is most apparent when comparing young with elderly individuals. The lack of age dependency observed in Schmid Daners et al. (2012) study may have been caused by a limited sample size.

In our study, age and sex only explain a small part of the variability of values of CSF flow dynamics parameters in the aqueduct as demonstrated by the adjusted *R*^2^ of the linear regression models. Yet this is not surprising as CSF flow in the aqueduct may be impacted by various factors such as biochemical parameters or cerebrovascular and brain pulsation (Mase et al., 1998; Chiang et al., 2009; Schmid Daners et al., 2012; Puy et al., 2016; Attier-Zmudka et al., 2019). Nonetheless between 6 and 18% of the variability in all parameters may be explained only by the two factors sex and age.

The reasons for sex and age dependencies of CSF flow are unknown to us, but we speculate that CSF volume regulation and thus CSF flow may be impacted by hormones and neural systems. The volume and osmolality of body fluids in general is regulated by neural and hormonal systems. It has been shown that estrogens and progestogens can influence both systems and it is known that certain neurons regulating fluid osmolality operate differently in males and females. During adulthood, hormone concentrations change, as observed after menopause in women, thus impacting an individual’s ability to regulate body fluid. For example, older women maintain thirst sensitivity to osmotic stimuli but lose sensitivity to changes in central body fluid volume. Moreover, older adults are more at risk of dehydration as they consume fluids at a slower rate and thus older individuals may have a lower central body fluid volume (Stachenfeld, 2014). The impact of these factors on the regulation of CSF volume and flow should be investigated in further studies.

Cerebrospinal fluid flow values are an interesting clinical predictor of conditions such as Chiari malformation or NPH (Bradley et al., 1996; Alperin et al., 2001; Luetmer et al., 2002; Stoquart-El Sankari et al., 2009; Gulbiz Kartal and Algin, 2014; Bradley, 2015; Markenroth Bloch et al., 2018). Specifically, while still largely debatable, there is evidence that flow velocity may be increased within the whole aqueduct in NPH (Bradley, 2015; Yamada et al., 2015). Furthermore, a net retrograde aqueductal flow (Ringstad et al., 2016; Lindstrøm et al., 2018) and an increased aqueductal stroke volume (Baroncini et al., 2018) are observed in NPH. However, aqueductal stroke volume does not reflect intracranial pressure pulsatility or symptom score, but rather aqueduct area and ventricular volume and therefore aqueductal stroke volume should not be used for selecting patients for shunting (Ringstad et al., 2015).

To exclude subjects with NPH we used the age-dependent Evans index (Brix et al., 2017), as it has been shown to be a useful tool to identify patients with NPH. However, one must acknowledge that the range of the normal Evans index is wide and thus its usage may be misleading on a case-by-case basis. We thus may have introduced a bias by possibly excluding participants with high Evans index values that may still have been normal. The Evans index can, however, not be used to distinguish healthy subjects from subjects with Alzheimer’s disease or with other diseases leading to cognitive impairment.

Interestingly, a very recent study (Attier-Zmudka et al., 2019) has observed decreased CSF flow in elderly patients with cognitive deficits whereby ventricular CSF flow was significantly associated with patient’s performance on an instrumental cognition test.

In our study we did not test subjects’ cognitive performance by means of neuropsychological testing but rather relied on neuroradiological metrics (MTA score) derived from morphological T_1_w images to evaluate hippocampal atrophy (Scheltens et al., 1992; Barkhof et al., 2007) and subjects with pathological MTA score were excluded from the study. Thus, subjects suffering from mild cognitive impairment and Alzheimer’s disease could be excluded. However, neuropsychological testing (such as the Mini-Mental State Examination; Kujawski et al., 2018; Herrmann et al., 2019; Hou et al., 2019; Santaella et al., 2019) may have been more reliable in assessing the cognitive status of participants. Therefore, we may have failed to exclude certain patients with mild cognitive impairment thus introducing a bias.

To date CSF flow values are not routinely used in a clinical setting, mainly caused by a lack of reliable reference values. Thus, when establishing reference values in future studies we recommend that the effect of age and sex should be considered. Specifically, reference values should be reported separately for males and females and for at least two age categories, especially in the parameters that are most influenced by sex and age (forward flow volume and absolute stroke volume).

Many parameters can be used to describe CSF flow in the aqueduct. One common parameter is peak pressure gradient. Optimally, this parameter is computed with the Navier–Stokes equation for incompressible and Newtonian fluids, as there are terms for viscosity and inertia in the equation. As performed in our study, the pressure gradient can, however, also be estimated with the simplified Bernoulli equation (Table 2) that assumes that viscous and inertial forces are negligible. While the simplified Bernoulli equation may suffice to characterize pressure gradients in subjects without obstructed subarachnoid spaces, it has been shown that pressure gradients calculated with the more precise Navier–Stokes equation deviate greatly from values obtained with the simplified Bernoulli equation in patients with obstructions. This should be taken into account when applying the results of this study to patients presenting with obstructions (Støverud et al., 2013; Ringstad et al., 2017).

A further parameter we quantified, that is less common in the field of CSF flow dynamics, is regurgitant fraction. It is routinely used in cardiology to assess whether heart valves function properly. It can be calculated by dividing the backward flow by the forward flow (Table 2). In case of CSF flow it may be useful to quantify the oscillating flow of CSF occurring in the aqueduct. However, it remains to be seen if this parameter is useful to diagnose pathological conditions affecting CSF flow.

When quantifying fluid flow, the parameters of the MR PC sequence play an important role. The flow rate of CSF within the aqueduct is very low and thus accurate quantification is a challenge (Najafi et al., 2018). We used an MR PC sequence that was able to quantify low flow rates with a maximum underestimation of 5–10% (Najafi et al., 2018). The *in vivo* CSF flow encountered in this study was sometimes, however, even lower than the minimum flow tested *in vitro* (0.1 ml/s) and thus the underestimation may have been slightly larger *in vivo*. One important parameter of the PC sequence is the VENC. The value of the VENC should be chosen as close to the velocity of the fluid flow encountered in subjects. We chose a relatively high VENC (22 cm/s) even though subjects presented with peak velocities of around 15 cm/s or less. While this may have caused a loss of accuracy in measuring the velocity of very low CSF flow it allows us to use the same VENC when studying patients presenting with pathologically elevated CSF flow (i.e., elevated velocity of fluid flow) thus enhancing reproducibility and usability of the results of this study.

Finally further limitations must be acknowledged. It is known that the magnitude of the aqueductal stroke volume is linked to the ventricular morphology if no aqueductal obstruction is present (Chiang et al., 2009). Aqueductal CSF flow is correlated with the total ventricular volume and the third ventricle width and volume (Brix et al., 2017). We did not measure the width and volume of the third ventricle. We just assumed a normal ventricular volume by excluding subjects with an Evans index of below 0.28 in subjects aged 50 years or younger and below 0.31 in subjects aged 51 years or older. Additionally, the effects of respiration on CSF flow parameters were not studied and were thus not considered. While respiration decisively impacts CSF flow, it has been shown that respiratory effects are averaged out when using conventional cardiac-gated PC MRI (Lindstrøm et al., 2018).

## Conclusion

Cerebrospinal fluid flow dynamics parameters as measured in the cerebral aqueduct are partially age and sex dependent. For establishment of reliable reference values in future studies, the impact of sex and age should be considered and incorporated.

## Data Availability

All data are available as the Supplementary Information.

## Author Contributions

TS, MW, ES, AN, and SS-S designed the study and interpreted the results. TS, MW, ES, AN, and SS-S performed the experiments. TS, MW, CR, and AN analyzed the data. TS, SS-S, and MW wrote the manuscript. CB and NH provided the technical advice. NG and TS conducted the statistical analysis. All coauthors contributed constructively to the manuscript.

## Conflict of Interest Statement

MW is a part time employee of Philips Healthcare, Switzerland. The remaining authors declare that the research was conducted in the absence of any commercial or financial relationships that could be construed as a potential conflict of interest.

## Abbreviations

CI: confidence interval
CSF: cerebrospinal fluid
3D: three dimensional
DRIVE: driven equilibrium
LPC: local phase correction
PC: phase contrast
PCA/P: phase of the phase contrast image
ROI: region of interest
SD: standard deviation
TFE: turbo field echo
TSE: turbo spin echo
T_1_w: T_1_-weighted
T_2_w: T_2_-weighted
VENC: velocity encoding.
