# Supplementary material for: Sex and Age Dependencies of Aqueductal Cerebrospinal Fluid Dynamics Parameters in Healthy Subjects
Source: Front Aging Neurosci. 2019 Aug 2;11:199. doi: 10.3389/fnagi.2019.00199 (PMC6688190; doi:10.3389/fnagi.2019.00199)
Supplement: Supplementary file 2 [file Data_Sheet_2.pdf]

Supplementary Figure

Scatter plot visualizing the heart rate. A regression line for the formula heart rate ~ age for both men and women was added with corresponding 95% confidence intervals (gray zones).

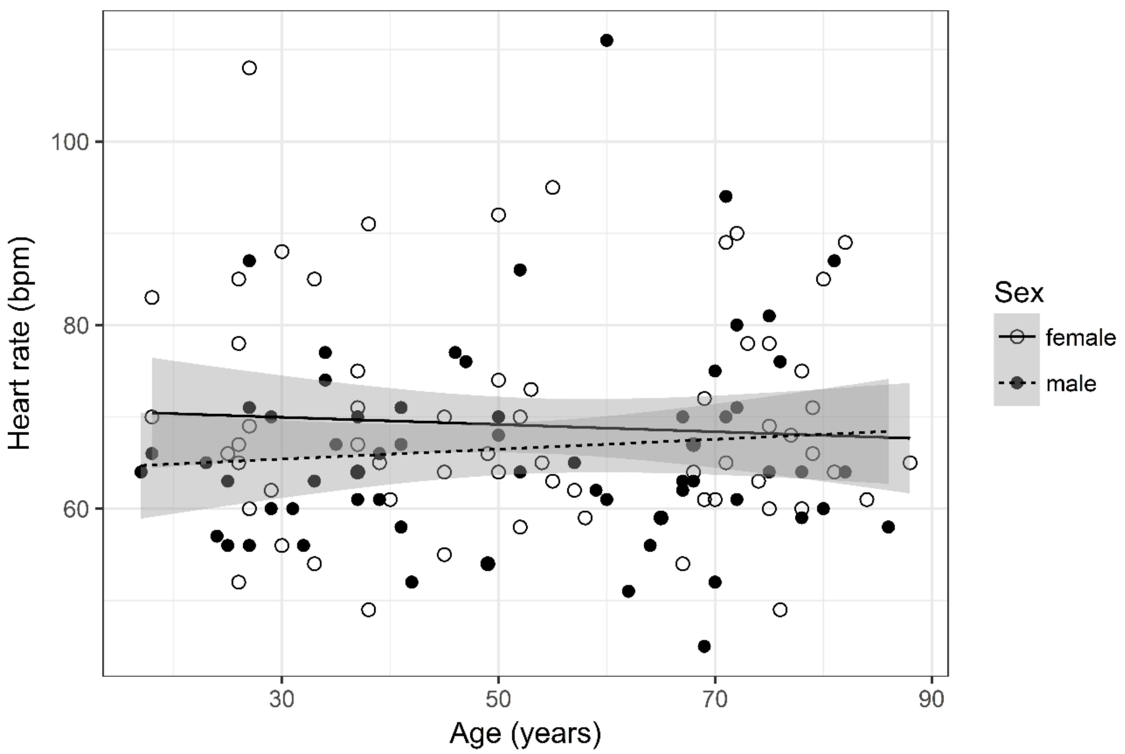

Supplementary Table

Sequence parameters of the sagittal 3D T2w TSE DRIVE sequence.

Parameters	3D T2w TSE DRIVE sequence
Field of View (FoV)	180 x 180mm^2
Acquired voxel size	0.6 x 0.6 x 0.6mm^3
Reconstructed voxel size	0.3 x 0.3 x 0.3mm^3
Number of slices	83
Repetition time (TR)	1500ms
Echo time (TE)	148ms
TSE Factor	45
SENSE factor	2
Number of signal averages (NSA)	1
Receiver bandwidth	381 Hz/pixel
Acquisition time [mm:ss]	05:39

Additionally as part of the supplementary material, a full statistical report (Data Sheet 3) and the raw data (large excel file, Data Sheet 1) of the CSF flow dynamics parameters is given.
