# Supplementary material for: Sex and Age Dependencies of Aqueductal Cerebrospinal Fluid Dynamics Parameters in Healthy Subjects
Source: Front Aging Neurosci. 2019 Aug 2;11:199. doi: 10.3389/fnagi.2019.00199 (PMC6688190; doi:10.3389/fnagi.2019.00199)
Supplement: Supplementary file 3 [file Data_Sheet_3.pdf]

graf biostatistics • Amelenweg 5 • 8400 Winterthur
phone 052 202 83 14 • mobile 079 270 67 72
graf@biostatistics.ch • www.biostatistics.ch

CSF Flow

Statistical Report

Nicole Graf (graf@biostatistics.ch)

Version 1.0 of 16/01/2019

- Data
- Statistical analysis
- Software
- Data visualisation
  - stroke volume
  - Forward flow vol
  - Backward flow vol
  - Regurgitant fract
  - Ab stroke volume
  - Mean flux
  - stroke distance
  - Mean velocity
  - Peak velocity
  - Peak pressure gradient
- Results
  - stroke volume
  - Forward flow vol
  - Backward flow vol
  - Regurgitant fract
  - Ab stroke volume
  - Mean flux
  - stroke distance
  - Mean velocity
  - Peak velocity
  - Peak pressure gradient

Create PDF in your applications with the Pdfcrowd HTML to PDF API PDFCROWD

- References

Data

This analysis is based on the data file “Daten_CSFFlow_07.01.2019_ASB.xlsm” received on January 9, 2019, via e-mail.

Statistical analysis

The ten endpoints were analysed with linear or ordinal regression models with predictors age and sex. For linear models, linearity was checked with component+residual plots. Homoscedasticity was checked with residual versus fitted values plot and tested with the standardized Breusch-Pagan test. Normality was checked with a Q-Q plot of the standardized residuals. For ordinal models, the proportional odds assumption was checked with a test of nominal effects and with a graphic by stratifying on each predictor and computing the logits of all proportions of the form Y>=j, j=1,2,…,k.

Software

All analyses were performed in the R programming language (version 3.3.3) (R Core Team, 2017). The package “MASS” (Venables and Ripley, 2002) was used to compute the ordinal regression models.

Data visualisation

stroke volume

Stroke volume has only very few values, thus, it cannot be treated as continuous variable. Stroke volume was recoded into four categories, i.e., 0, 0.01, 0.02, and >=0.03.

Create PDF in your applications with the Pdfcrowd HTML to PDF API PDFCROWD

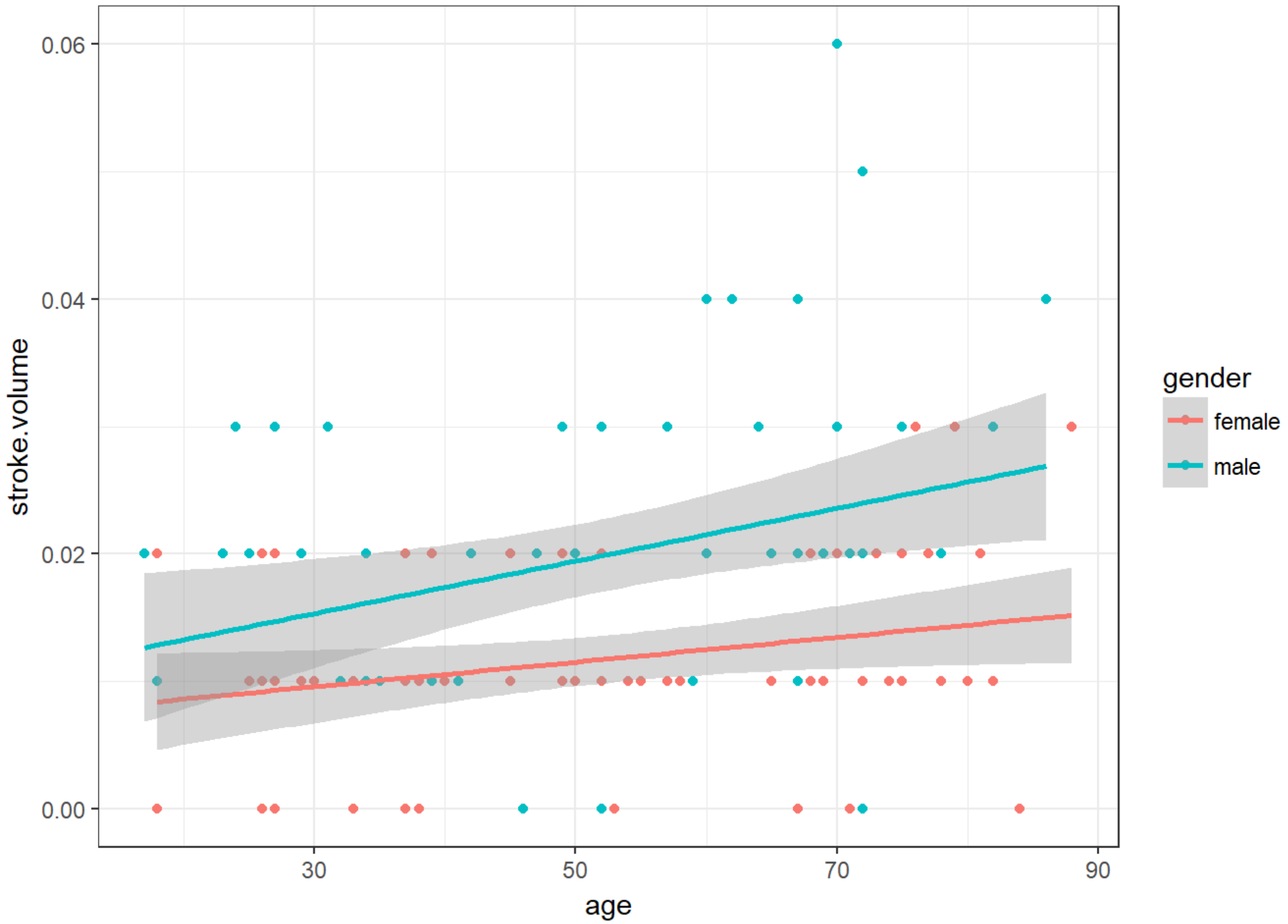

Figure 1. Scatterplot between age and stroke.volume. The regression line stroke.volume ~ age for male and female probands was added.

Forward flow vol

Create PDF in your applications with the Pdfcrowd HTML to PDF API PDFCROWD

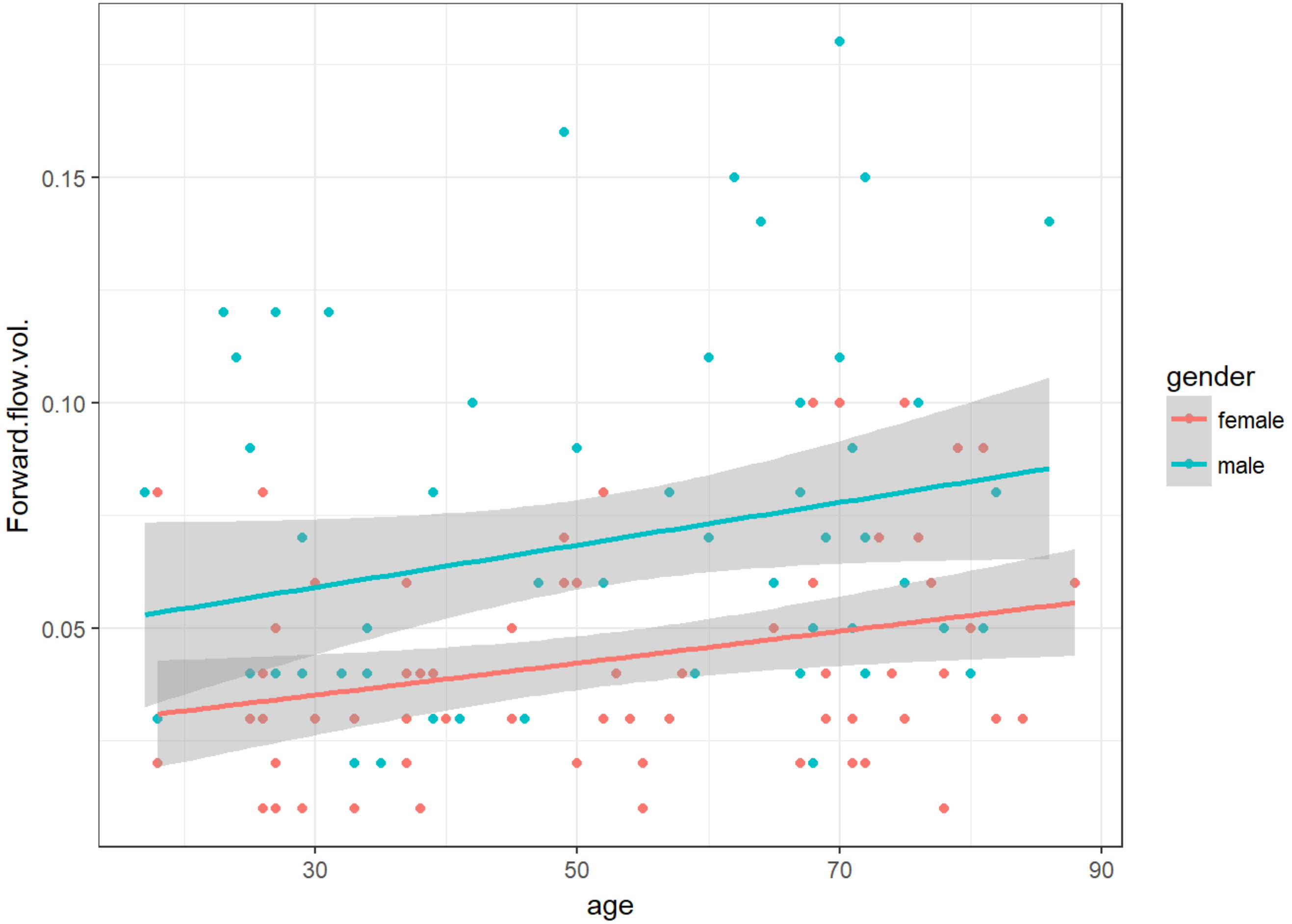

Figure 2. Scatterplot between age and Forward.flow.vol. The regression line Forward.flow.vol. ~ age for male and female probands was added.

Backward flow vol

Create PDF in your applications with the Pdfcrowd HTML to PDF API PDFCROWD

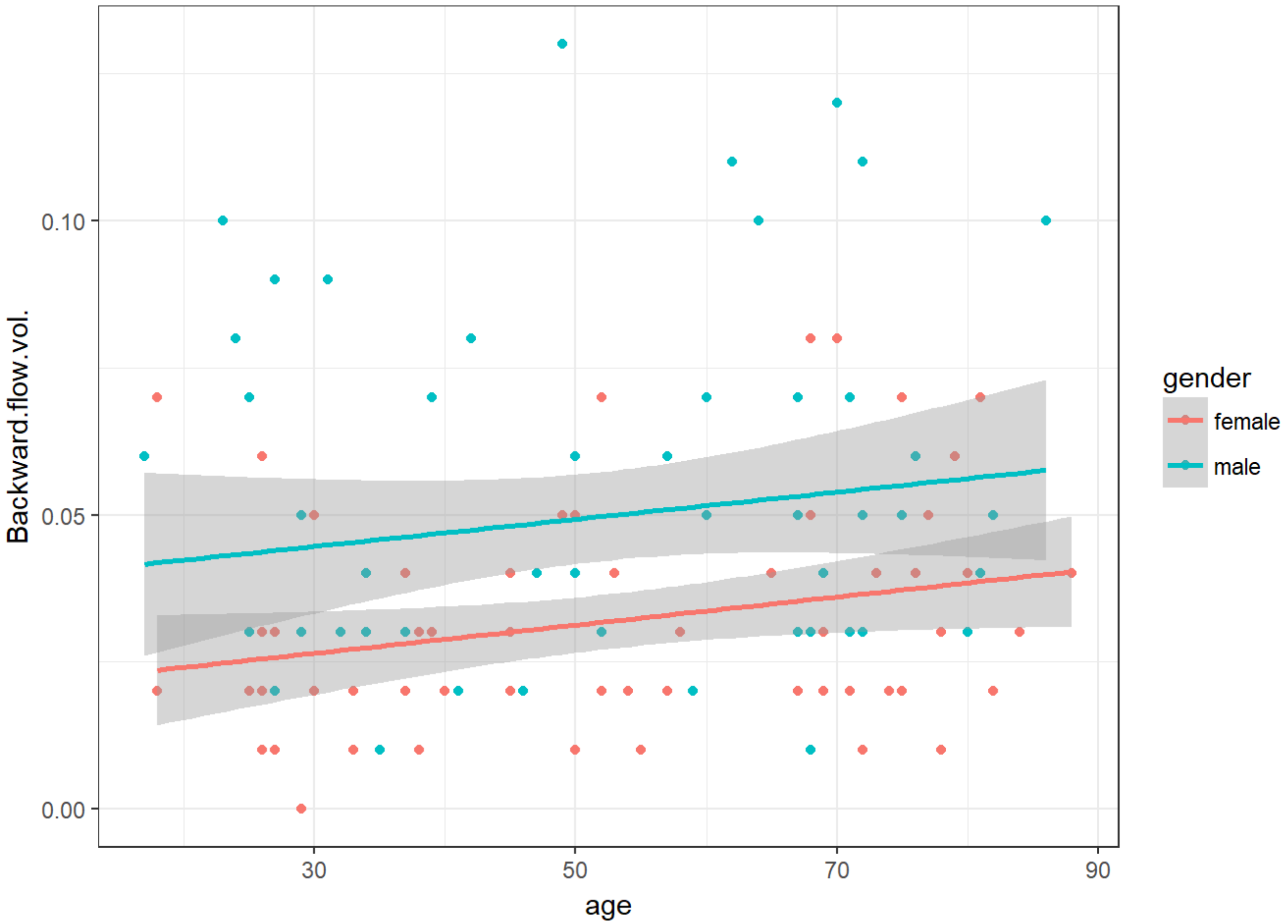

Figure 3. Scatterplot between age and Backward.flow.vol. The regression line Backward.flow.vol. ~ age for male and female probands was added.

Regurgitant fract

Create PDF in your applications with the Pdfcrowd HTML to PDF API PDFCROWD

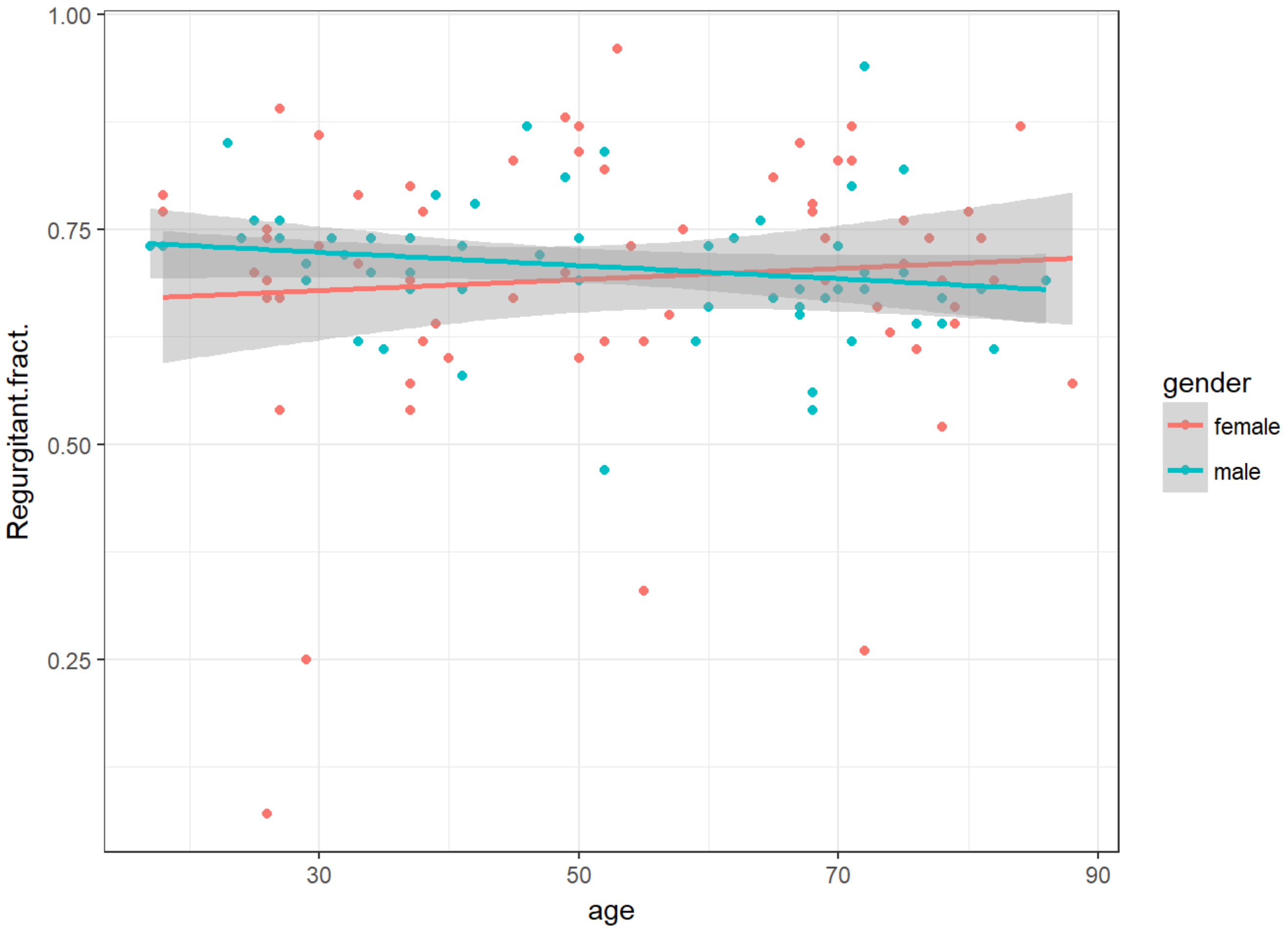

Figure 4. Scatterplot between age and Regurgitant.fract. The regression line Regurgitant.fract. ~ age for male and female probands was added.

Ab stroke volume

Create PDF in your applications with the Pdfcrowd HTML to PDF API PDFCROWD

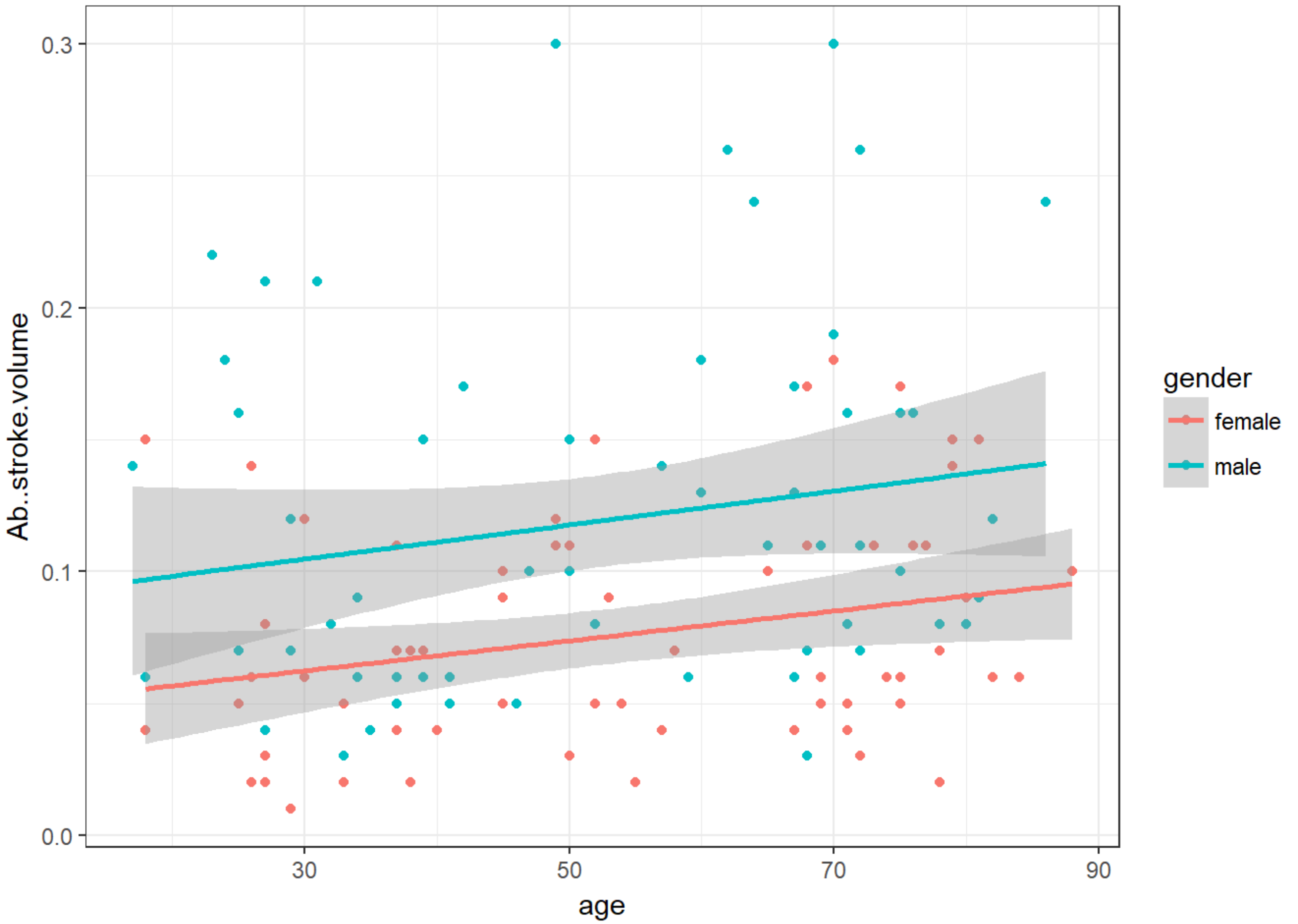

Figure 5. Scatterplot between age and Ab..stroke.volume. The regression line Ab..stroke.volume ~ age for male and female probands was added.

Mean flux

Mean flux has only very few values, thus, it cannot be treated as continuous variable. Mean flux was recoded into four categories, i.e., 0, 0.01, 0.02, and >= 0.03.

Create PDF in your applications with the Pdfcrowd HTML to PDF API PDFCROWD

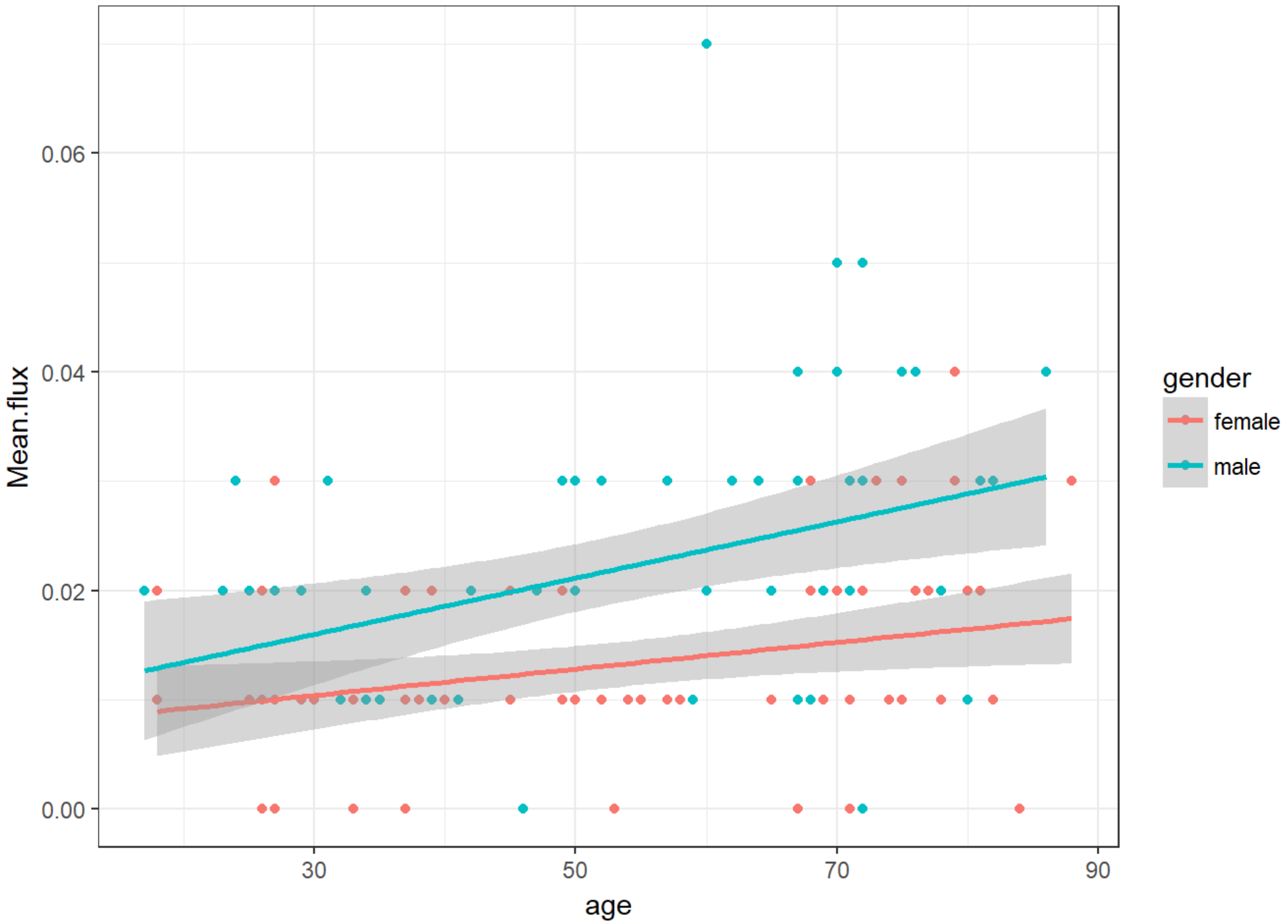

Figure 6. Scatterplot between age and Mean.flux. The regression line Mean.flux ~ age for male and female probands was added.

stroke distance

Create PDF in your applications with the Pdfcrowd HTML to PDF API PDFCROWD

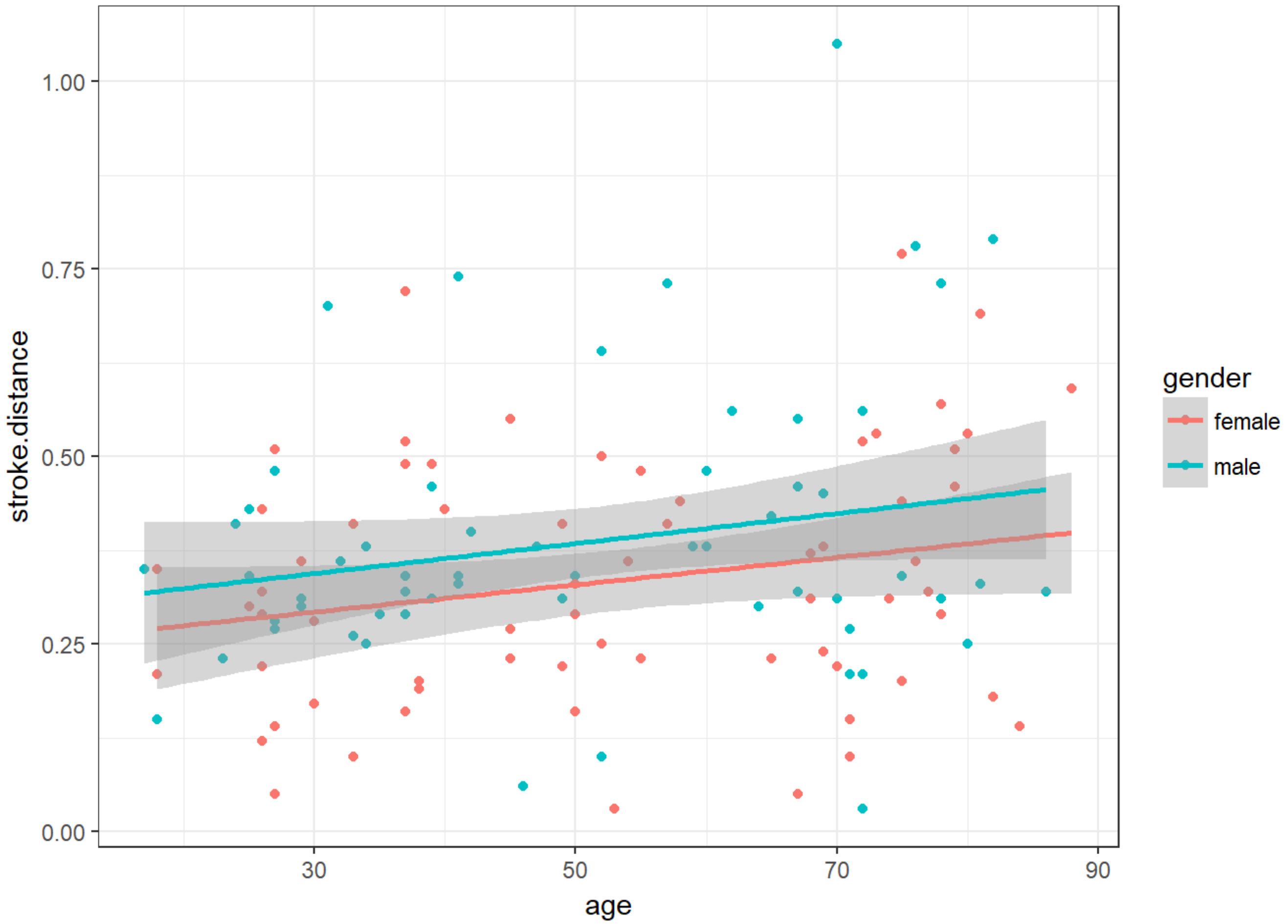

Figure 7. Scatterplot between age and stroke.distance. The regression line stroke.distance ~ age for male and female probands was added.

Mean velocity

Create PDF in your applications with the Pdfcrowd HTML to PDF API PDFCROWD

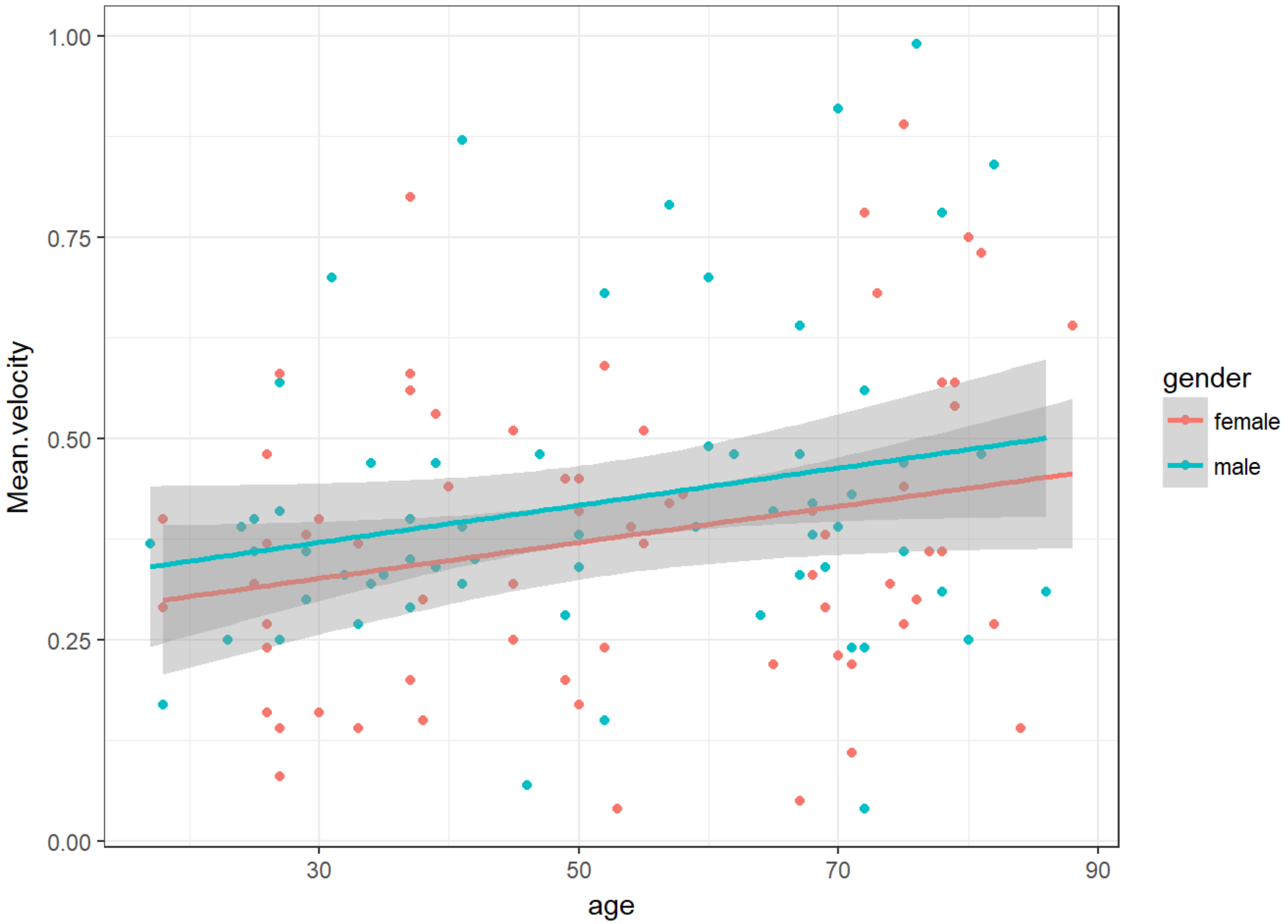

Figure 8. Scatterplot between age and Mean.velocity. The regression line Mean.velocity ~ age for male and female probands was added.

Peak velocity

Create PDF in your applications with the Pdfcrowd HTML to PDF API PDFCROWD

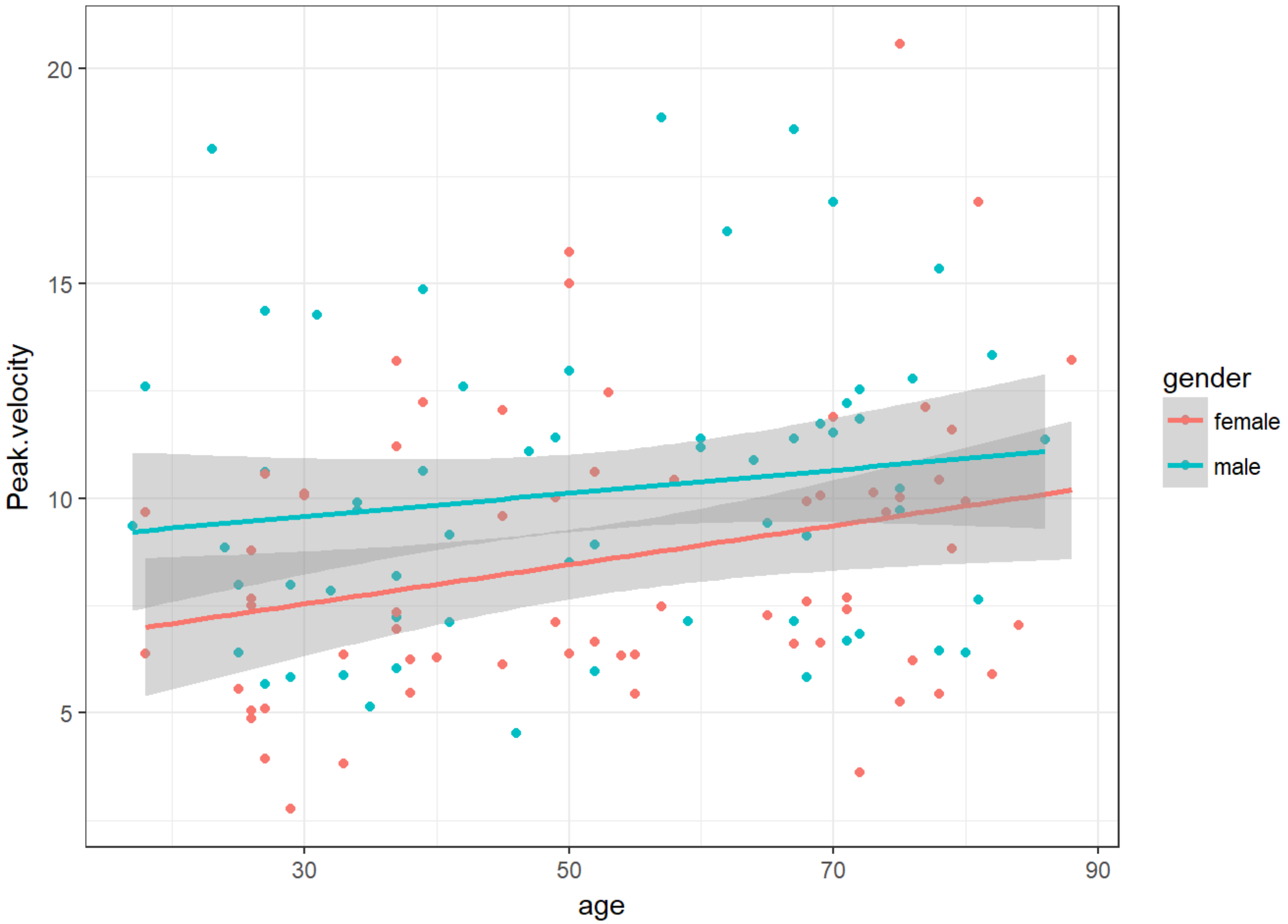

Figure 9. Scatterplot between age and Peak.velocity. The regression line Peak.velocity ~ age for male and female probands was added.

Peak pressure gradient

Create PDF in your applications with the Pdfcrowd HTML to PDF API PDFCROWD

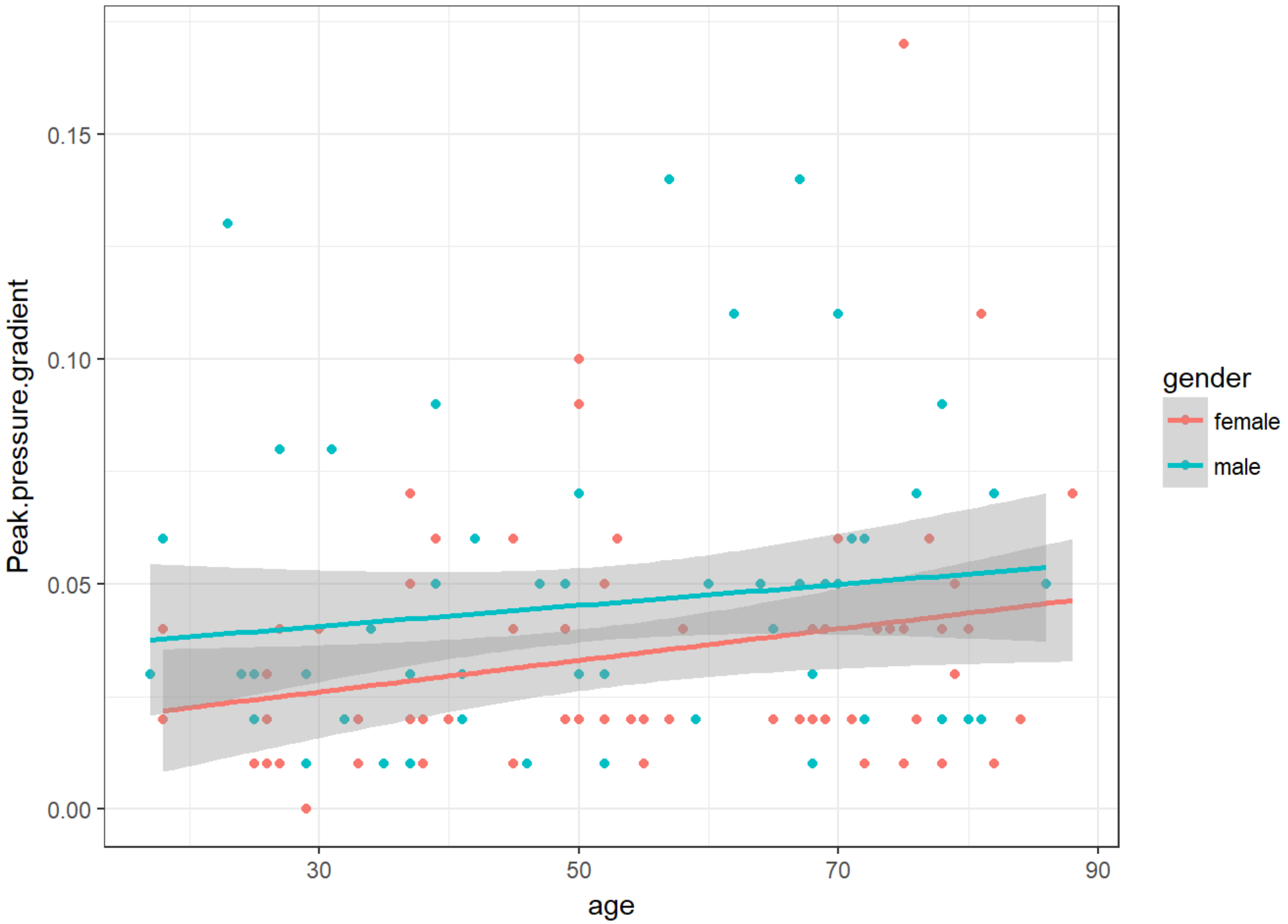

Figure 10. Scatterplot between age and Peak.pressure.gradient. The regression line Peak.pressure.gradient ~ age for male and female probands was added.

Results

stroke volume

Create PDF in your applications with the Pdfcrowd HTML to PDF API PDFCROWD

An ordinal regression was calculated for stroke.volume that was recoded into a variable with four categories. The test of nominal effects indicated that the proportional odds assumption was met. Furthermore, the distances between the symbols for each set of categories of the dependent variable remained fairly similar (Fig. 11). The first set of coefficients was set to zero to have a common reference point.
The ordinal regression indicates that both gender ($p<0.001$) and age ($p=0.003$) are significant predictors for stroke.volume. As age increases by one unit, the odds of observing category 4 of stroke.volume vs. the other 3 categories increase by a factor of 1.03. The odds of observing category 4 of stroke.volume vs. the other 3 categories are 4.59 times higher for male than for female probands.

```
## Call:
## polr(formula = stroke.volume.rec ~ gender + age, data = mydata,
##     Hess = TRUE)
##
## Coefficients:
##              Value Std. Error t value
## gendermale 1.52453   0.356511   4.276
## age        0.02666   0.008847   3.013
##
## Intercepts:
##     Value   Std. Error t value
## 1|2 -0.2693  0.5281    -0.5099
## 2|3  2.3512  0.5503     4.2724
## 3|4  4.0101  0.6305     6.3604
##
## Residual Deviance: 294.1971
## AIC: 304.1971
```

```
##                  Value  Std. Error    t value      p value
## gendermale  1.5245307 0.356510723  4.2762547 1.900638e-05
## age         0.0266615 0.008847408  3.0134816 2.582687e-03
## 1|2        -0.2693161 0.528131656 -0.5099412 6.100927e-01
## 2|3         2.3511615 0.550312344  4.2724128 1.933691e-05
## 3|4         4.0101414 0.630488432  6.3603727 2.012648e-10
```

```
##                   OR    2.5 %   97.5 %
## gendermale 4.592987 2.314276 9.395574
## age        1.027020 1.009601 1.045320
```

Create PDF in your applications with the Pdfcrowd HTML to PDF API PDFCROWD

```
## as.numeric(stroke.volume.rec)    N=128
##
## +-------+-------+---+----+----+---------+---------+
## |       |       |N  |Y>=1|Y>=2|Y>=3     |Y>=4     |
## +-------+-------+---+----+----+---------+---------+
## |gender |female | 66|Inf |0   |-2.515147|-4.350278|
## |       |male   | 62|Inf |0   |-2.519393|-3.952374|
## +-------+-------+---+----+----+---------+---------+
## |age    |[17,35)| 32|Inf |0   |-2.592537|-4.214594|
## |       |[35,53)| 35|Inf |0   |-2.573786|-4.851053|
## |       |[53,72)| 32|Inf |0   |-1.945910|-3.218876|
## |       |[72,88]| 29|Inf |0   |-2.254383|-3.401197|
## +-------+-------+---+----+----+---------+---------+
## |Overall|       |128|Inf |0   |-2.316770|-3.725448|
## +-------+-------+---+----+----+---------+---------+
```

Create PDF in your applications with the Pdfcrowd HTML to PDF API PDFCROWD

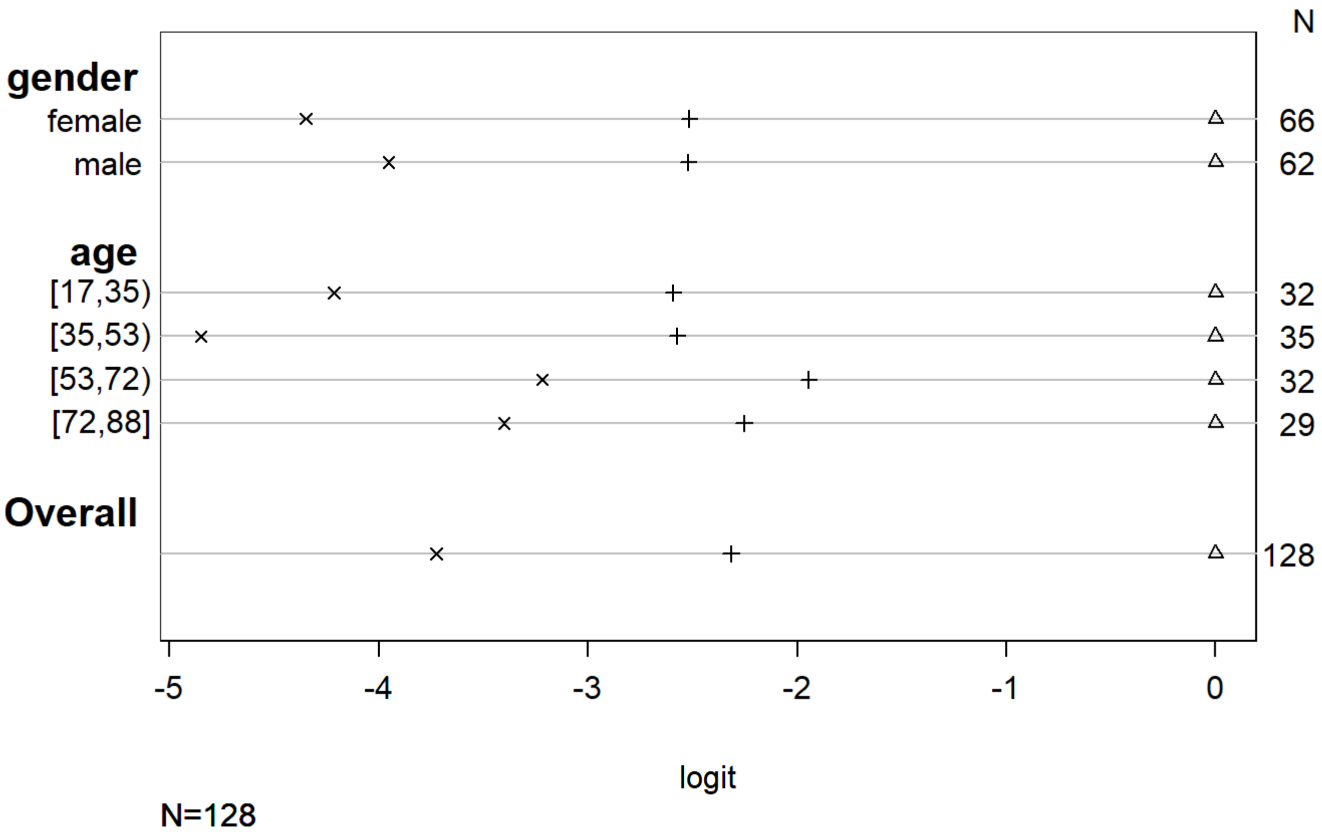

Figure 11. Checking proportional odds assumption. The cross, plus, and triangle sign correspond to Y >=4,3,2, respectively.

```
## Tests of nominal effects
##
## formula: stroke.volume.rec ~ gender + age
##        Df  logLik    AIC     LRT Pr(>Chi)
## <none>    -147.10 304.20
## gender  2 -146.90 307.80 0.40143   0.8181
## age     2 -145.77 305.54 2.65376   0.2653
```

Create PDF in your applications with the Pdfcrowd HTML to PDF API PDFCROWD

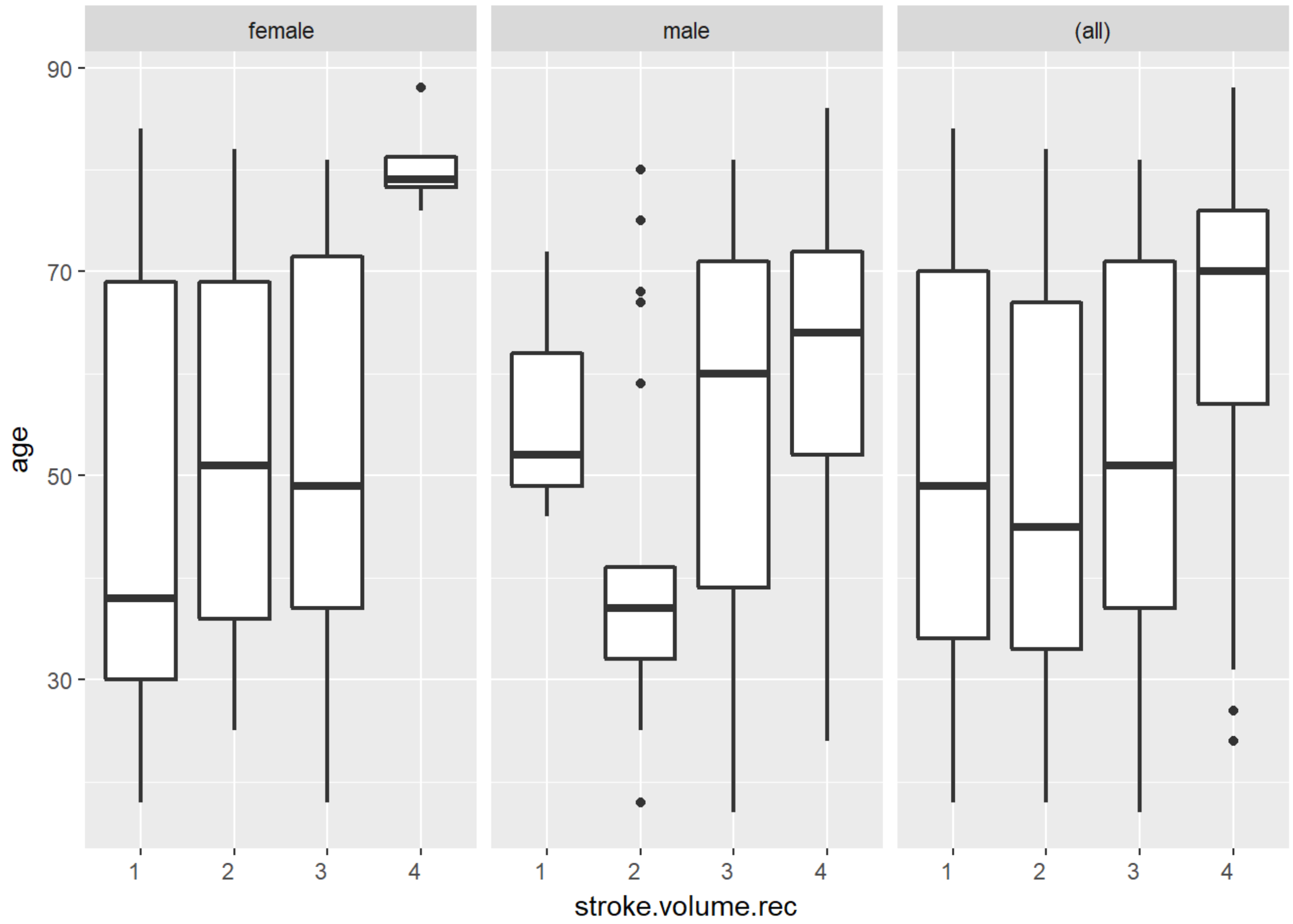

Figure 12. Boxplots for age for every level of stroke.volume and gender.

Forward flow vol

Model mod2a (Forward.flow.vol. ~ gender + age) did not meet assumption of homoscedasticity (see studentized Breusch-Pagan test). Therefore, Forward.flow.vol was logarithmized. Model 2b met all assumptions. Thus, age was fairly linearly related to Forward.flow.vol. (Fig. 13), assumptions of homoscedasticity (Fig. 14, residuals vs fitted values plot, scale-location plot, and Breusch-Pagan test) and normality (Fig. 14, QQ plot) were also met.

The analysis of variance table indicates that both gender ($p<0.001$) and age ($p=0.002$) are significant predictors for Forward.flow.vol. Values were

Create PDF in your applications with the Pdfcrowd HTML to PDF API PDFCROWD

higher for older compared to younger probands and male compared to female probands. (For log-transformed outcomes the results can be interpreted as percentage increase, i.e., male sex leads to 21.5% increase in Forward.flow.vol compared to female sex, or an increase of one year in age leads to a 0.36% increase in Forward.flow.vol.)

```
##
##  studentized Breusch-Pagan test
##
## data:  mod2a
## BP = 11.716, df = 1, p-value = 0.0006197
```

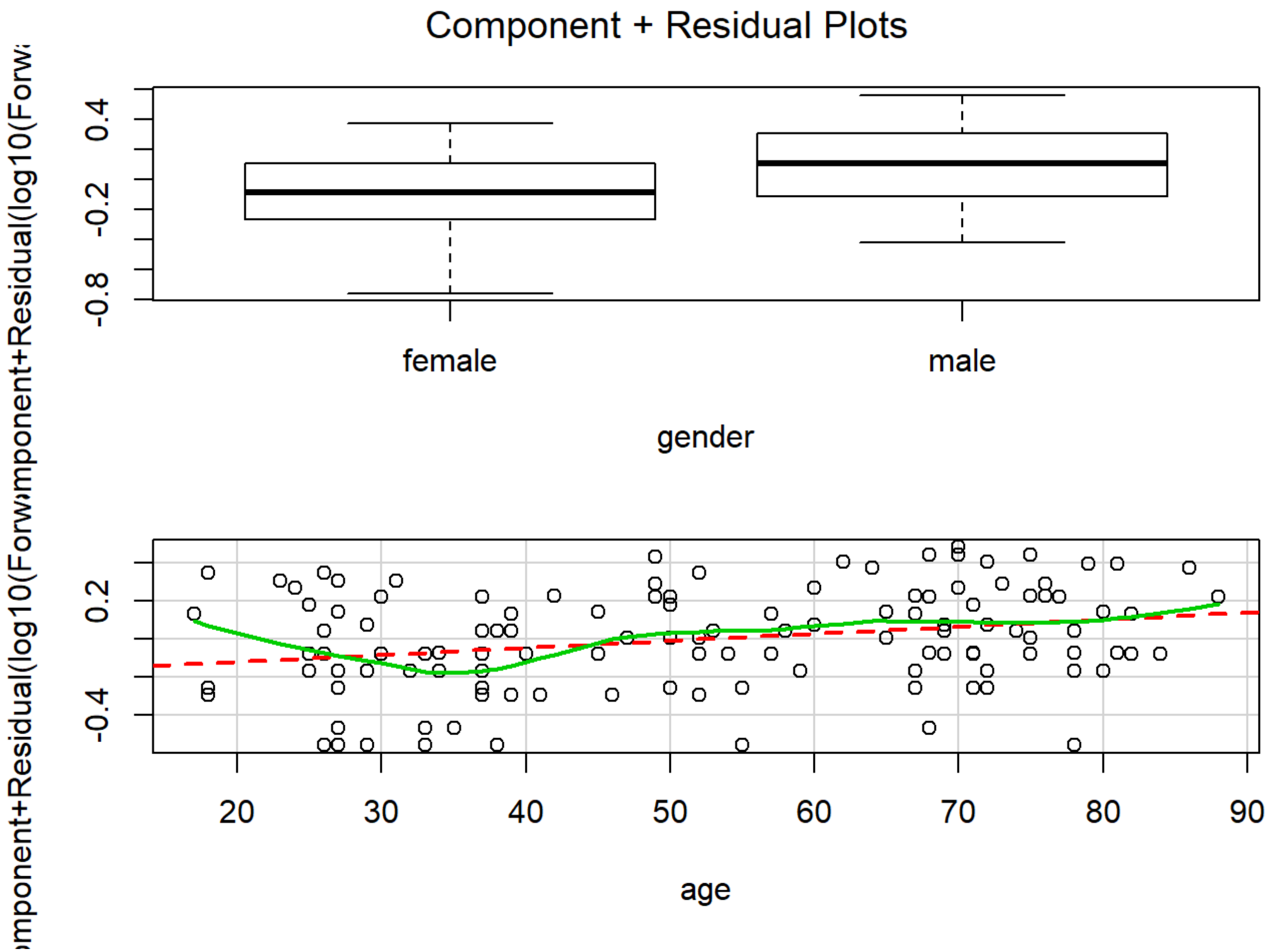

Create PDF in your applications with the Pdfcrowd HTML to PDF API PDFCROWD

Figure 13. Component+residual plot to check linearity.

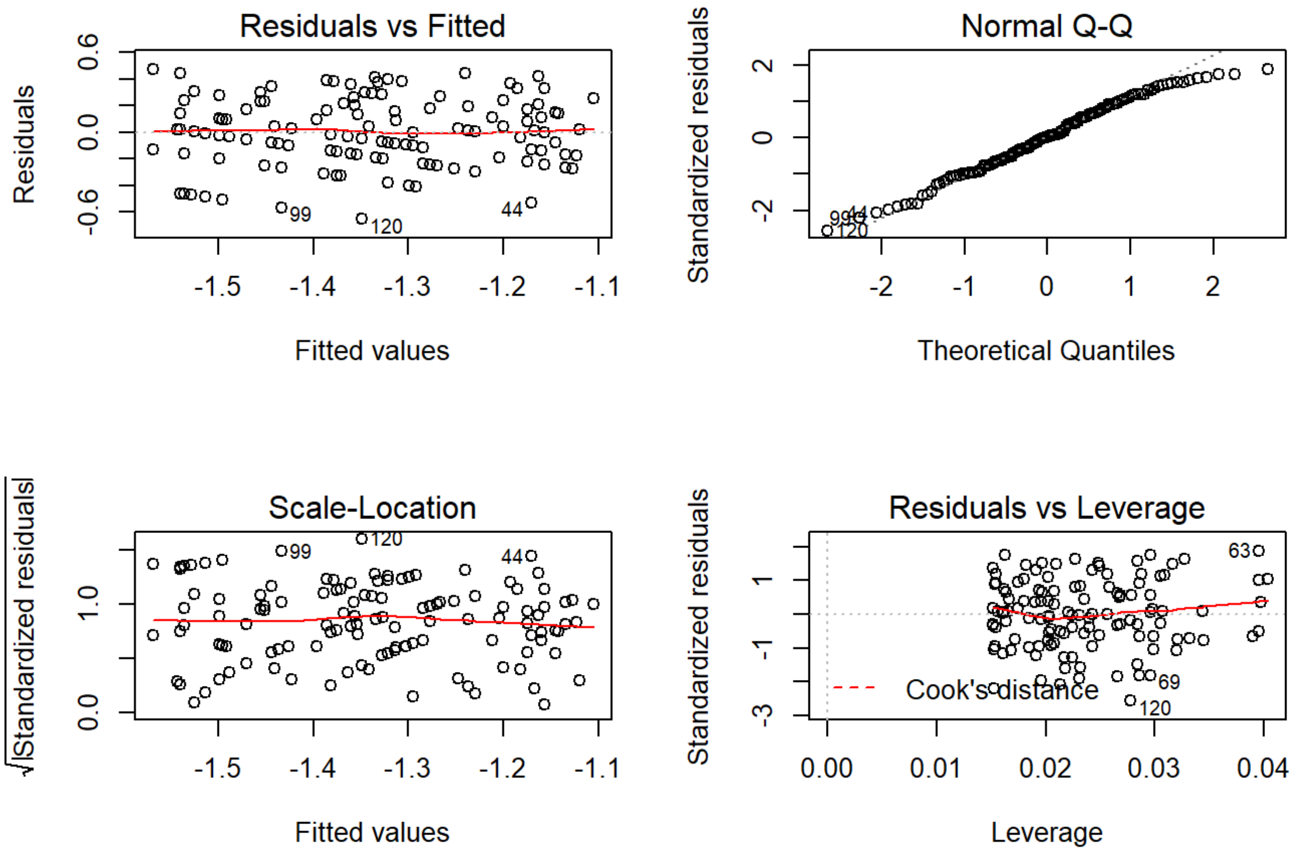

Figure 14. Plots to assess assumptions of homoscedasticity and normality.

```
##
##  studentized Breusch-Pagan test
##
## data:  mod2b
## BP = 1.8341, df = 1, p-value = 0.1756
```

Create PDF in your applications with the Pdfcrowd HTML to PDF API PDFCROWD

```
## Analysis of Variance Table
##
## Response: log10(Forward.flow.vol.)
##            Df Sum Sq Mean Sq F value    Pr(>F)
## gender      1 1.4220 1.42203  21.505 8.757e-06 ***
## age         1 0.6714 0.67143  10.154  0.001819 **
## Residuals 125 8.2657 0.06613
## ---
## Signif. codes:  0 '***' 0.001 '**' 0.01 '*' 0.05 '.' 0.1 ' ' 1
```

```
##
## Call:
## lm(formula = log10(Forward.flow.vol.) ~ gender + age, data = mydata)
##
## Residuals:
##      Min       1Q   Median       3Q      Max
## -0.65080 -0.18044  0.00487  0.20245  0.47208
##
## Coefficients:
##              Estimate Std. Error t value Pr(>|t|)
## (Intercept) -1.634925   0.068581 -23.839  < 2e-16 ***
## gendermale   0.215002   0.045498   4.725 6.07e-06 ***
## age          0.003663   0.001150   3.187  0.00182 **
## ---
## Signif. codes:  0 '***' 0.001 '**' 0.01 '*' 0.05 '.' 0.1 ' ' 1
##
## Residual standard error: 0.2571 on 125 degrees of freedom
## Multiple R-squared:  0.2021, Adjusted R-squared:  0.1893
## F-statistic: 15.83 on 2 and 125 DF,  p-value: 7.451e-07
```

```
##                    2.5 %      97.5 %
## (Intercept) -1.770656003 -1.499194253
## gendermale   0.124954760  0.305048384
## age          0.001387963  0.005938227
```

Create PDF in your applications with the Pdfcrowd HTML to PDF API PDFCROWD

Backward flow vol

Model mod3a (Backward.flow.vol. ~ gender + age) did not meet assumption of homoscedasticity (see studentized Breusch-Pagan test). Therefore, Backward.flow.vol was logarithmized (one zero value was set to missing). Model 2b met all assumptions. Thus, age was fairly linearly related to Backward.flow.vol. (Fig. 15), assumptions of homoscedasticity (Fig. 16, residuals vs fitted values plot, scale-location plot, and Breusch-Pagan test) and normality (Fig. 16, QQ plot) were also met.
The analysis of variance table indicates that both gender ($p<0.001$) and age ($p=0.018$) are significant predictors for Backward.flow.vol. Values were higher for older compared to younger probands and male compared to female probands.

```
##
##  studentized Breusch-Pagan test
##
## data:  mod3a
## BP = 8.439, df = 1, p-value = 0.003672
```

Create PDF in your applications with the Pdfcrowd HTML to PDF API PDFCROWD

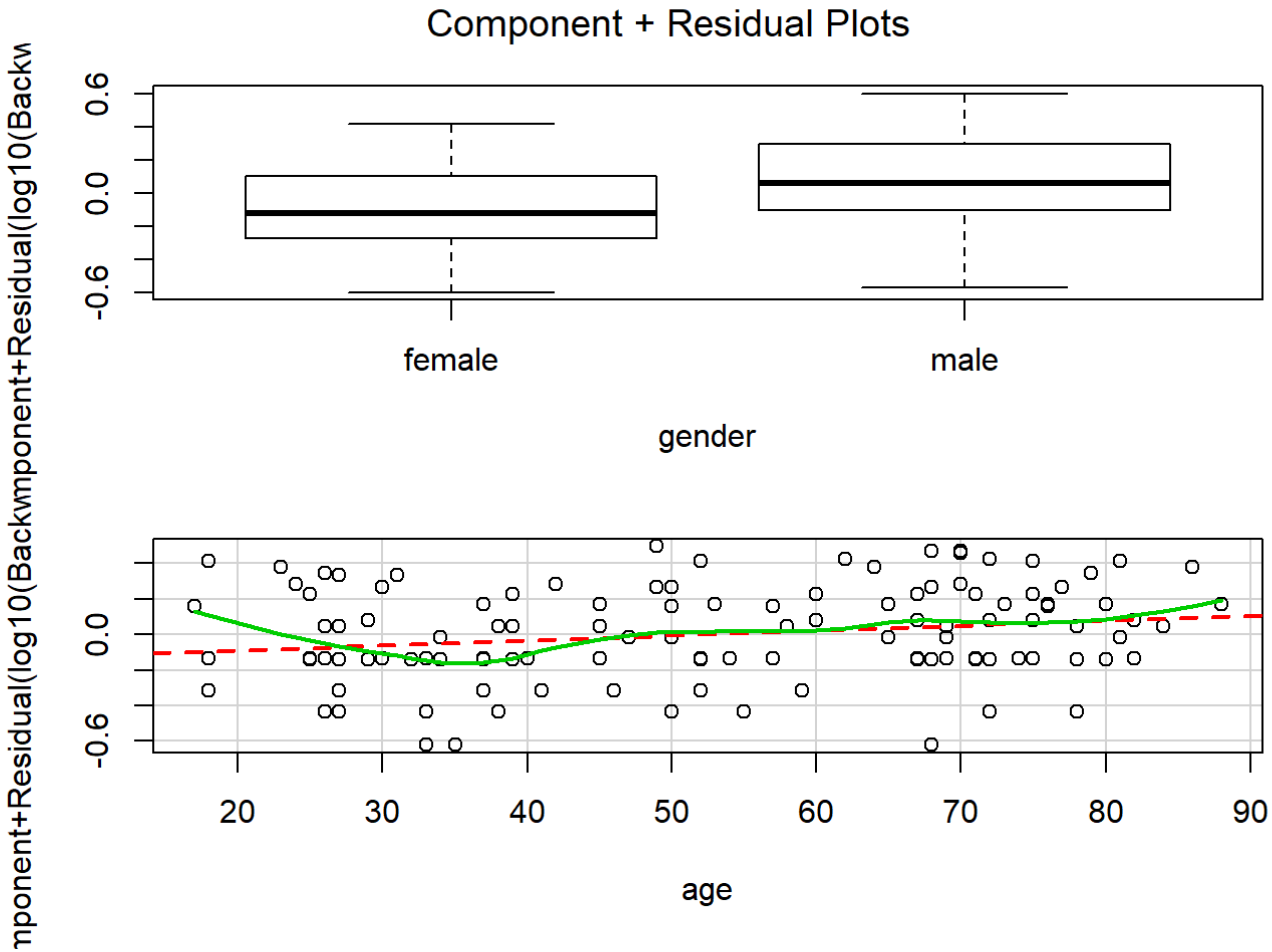

Figure 15. Component+residual plot to check normality.

Create PDF in your applications with the Pdfcrowd HTML to PDF API PDFCROWD

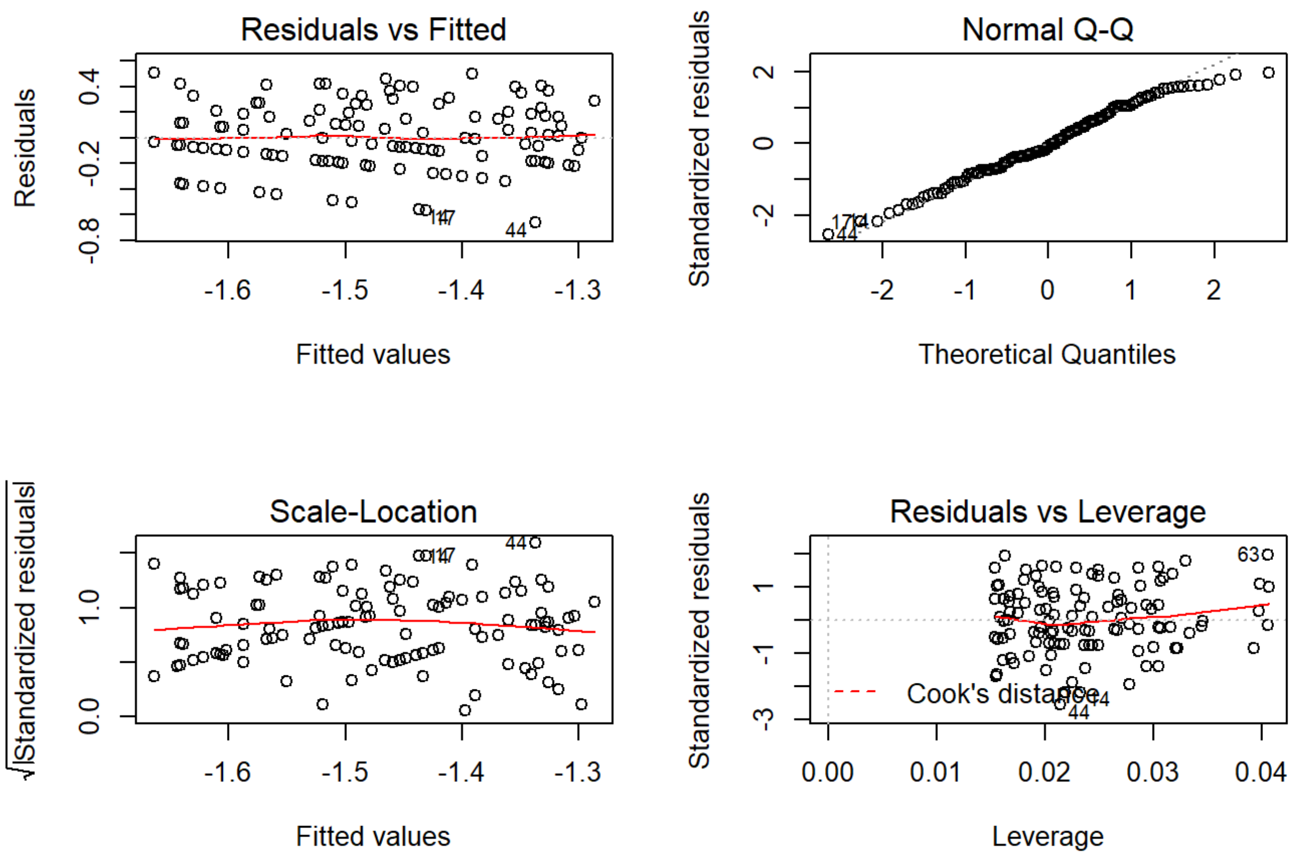

Figure 16. Plots to assess assumptions of homoscedasticity and normality.

```
##
##  studentized Breusch-Pagan test
##
## data:  mod3b
## BP = 0.046103, df = 1, p-value = 0.83
```

```
## Analysis of Variance Table
##
```

Create PDF in your applications with the Pdfcrowd HTML to PDF API PDFCROWD

```
## Response: log10(Backward.flow.vol.)
##             Df Sum Sq Mean Sq F value   Pr(>F)
## gender      1 1.0374 1.03742 14.9407 0.000178 ***
## age         1 0.3972 0.39722  5.7207 0.018268 *
## Residuals 124 8.6101 0.06944
## ---
## Signif. codes:  0 '***' 0.001 '**' 0.01 '*' 0.05 '.' 0.1 ' ' 1
```

```
##
## Call:
## lm(formula = log10(Backward.flow.vol.) ~ gender + age, data = mydata.rec)
##
## Residuals:
##      Min       1Q   Median       3Q      Max
## -0.66245 -0.18511 -0.02864  0.20068  0.50938
##
## Coefficients:
##              Estimate Std. Error t value Pr(>|t|)
## (Intercept) -1.715291   0.071103 -24.124  < 2e-16 ***
## gendermale   0.185023   0.046811   3.953 0.000129 ***
## age          0.002834   0.001185   2.392 0.018268 *
## ---
## Signif. codes:  0 '***' 0.001 '**' 0.01 '*' 0.05 '.' 0.1 ' ' 1
##
## Residual standard error: 0.2635 on 124 degrees of freedom
## Multiple R-squared:  0.1428, Adjusted R-squared:  0.129
## F-statistic: 10.33 on 2 and 124 DF,  p-value: 7.084e-05
```

```
##                     2.5 %       97.5 %
## (Intercept) -1.8560233296 -1.574558605
## gendermale   0.0923703563  0.277675143
## age          0.0004887914  0.005179252
```

Regurgitant fract

Create PDF in your applications with the Pdfcrowd HTML to PDF API PDFCROWD

Model mod4a (Regurgitant.fract. ~ gender + age) did not meet assumption of homoscedasticity (see studentized Breusch-Pagan test). Therefore, Regurgitant.fract. was logarithmized. There was one extreme and influential outlier (66=KSWFlow_Vol_003), which was removed. Model mod4c met all assumptions. Thus, age was fairly linearly related to Regurgitant.fract. (Fig. 17), assumptions of homoscedasticity (Fig. 18, residuals vs fitted values plot, scale-location plot, and Breusch-Pagan test) and normality (Fig. 18, QQ plot) were also met.
The analysis of variance table indicates that neither gender (p=0.502) nor age (p=0.559) are significant predictors for Rugurgitant.flow. (The interaction between age and gender was also tested but did not prove to be significant.)

```
##
##  studentized Breusch-Pagan test
##
## data:  mod4a
## BP = 4.7999, df = 1, p-value = 0.02846
```

Create PDF in your applications with the Pdfcrowd HTML to PDF API PDFCROWD

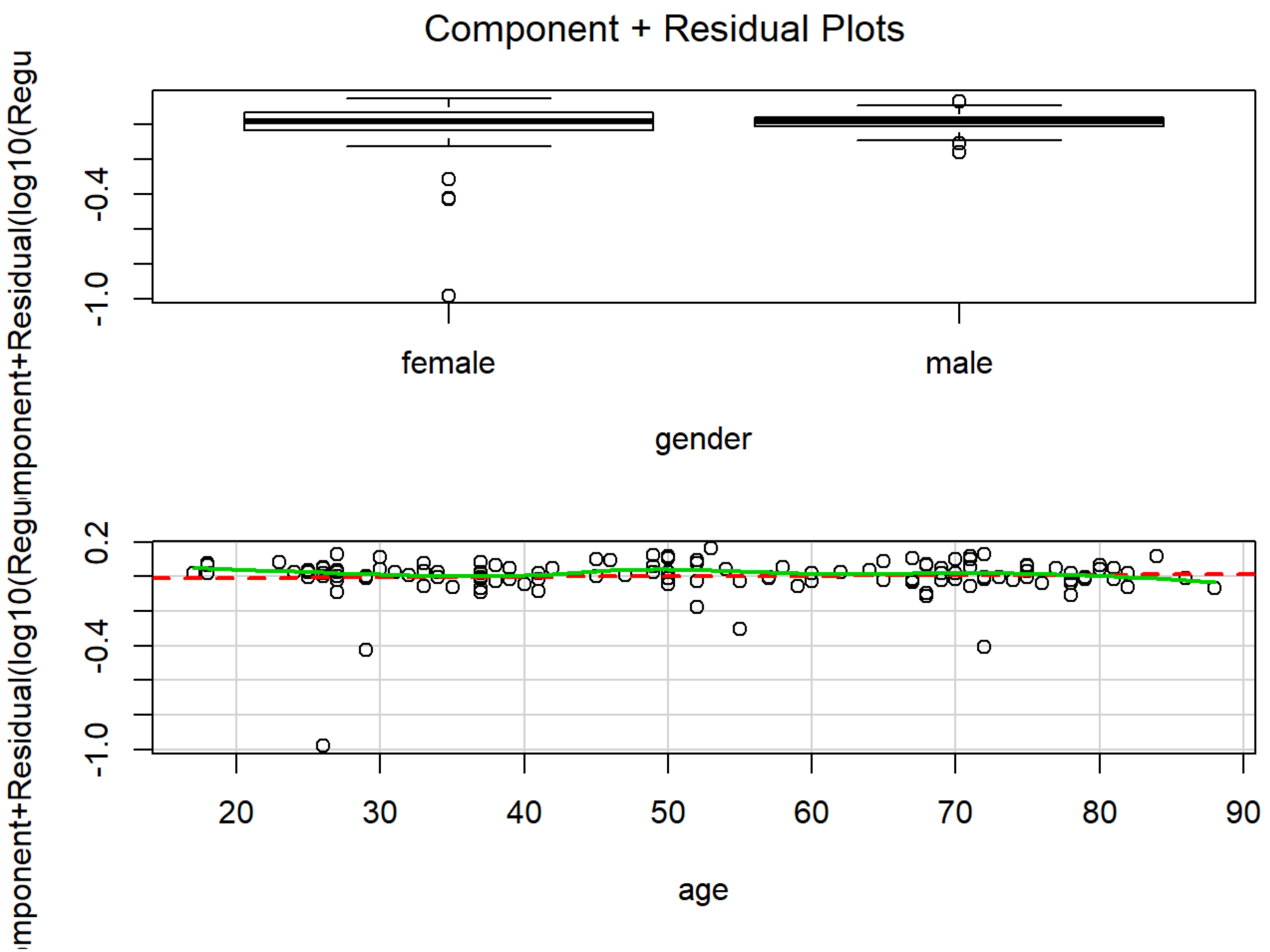

Figure 17. Component+residual plot to check linearity.

Create PDF in your applications with the Pdfcrowd HTML to PDF API PDFCROWD

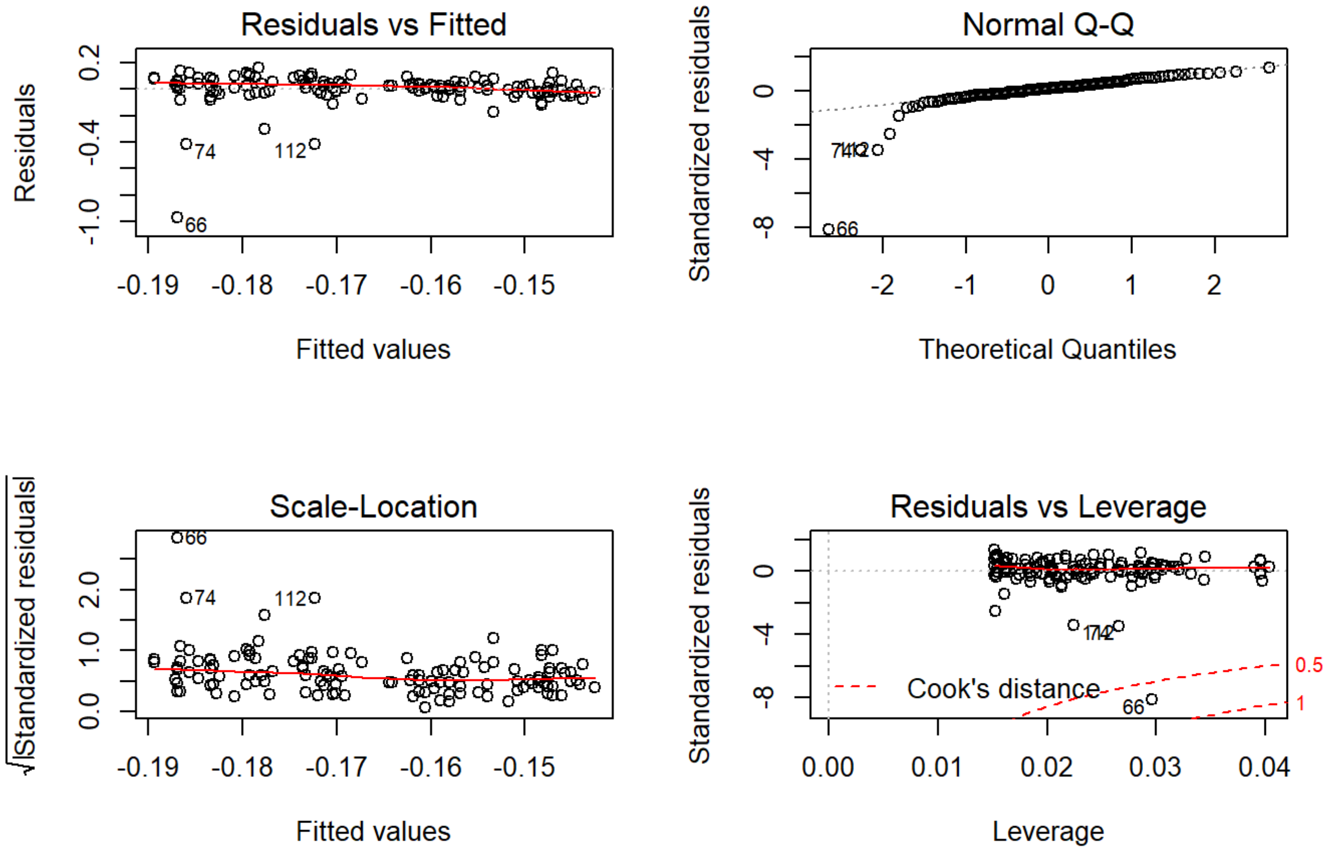

Figure 18. Plots to assess assumptions of homoscedasticity and normality.

```
##
##  studentized Breusch-Pagan test
##
## data:  mod4c
## BP = 2.0727, df = 1, p-value = 0.15
```

```
## Analysis of Variance Table
##
```

Create PDF in your applications with the Pdfcrowd HTML to PDF API PDFCROWD

```
## Response: log10(Regurgitant.fract.)
##            Df  Sum Sq   Mean Sq F value Pr(>F)
## gender      1 0.00315 0.0031486  0.4531 0.5021
## age         1 0.00239 0.0023907  0.3441 0.5586
## Residuals 124 0.86157 0.0069482
```

```
##
## Call:
## lm(formula = log10(Regurgitant.fract.) ~ gender + age, data = mydata.rec)
##
## Residuals:
##      Min       1Q   Median       3Q      Max
## -0.44407 -0.02333  0.00749  0.04470  0.14555
##
## Coefficients:
##               Estimate Std. Error t value Pr(>|t|)
## (Intercept) -0.1516033  0.0225353  -6.727 5.68e-10 ***
## gendermale   0.0096237  0.0148085   0.650    0.517
## age         -0.0002202  0.0003754  -0.587    0.559
## ---
## Signif. codes:  0 '***' 0.001 '**' 0.01 '*' 0.05 '.' 0.1 ' ' 1
##
## Residual standard error: 0.08336 on 124 degrees of freedom
## Multiple R-squared:  0.006388,   Adjusted R-squared:  -0.009638
## F-statistic: 0.3986 on 2 and 124 DF,  p-value: 0.6721
```

```
##                     2.5 %        97.5 %
## (Intercept) -0.1962069495 -0.1069995519
## gendermale  -0.0196865744  0.0389339603
## age         -0.0009632411  0.0005228292
```

Ab stroke volume

Create PDF in your applications with the Pdfcrowd HTML to PDF API PDFCROWD

Model mod5a (Ab..stroke.volume ~ gender + age) did not meet assumption of homoscedasticity (see studentized Breusch-Pagan test). Therefore, Ab..stroke.volume was logarithmized. Model mod5b met all assumptions. Thus, age was fairly linearly related to Ab..stroke.volume (Fig. 19), assumptions of homoscedasticity (Fig. 20, residuals vs fitted values plot, scale-location plot, and Breusch-Pagan test) and normality (Fig. 20, QQ plot) were also met.
The analysis of variance table indicates that both gender (p<0.001) and age (p=0.005) are significant predictors for Ab..stroke.volume. Values were higher for older compared to younger probands and male compared to female probands.

```
##
##  studentized Breusch-Pagan test
##
## data:  mod5a
## BP = 9.8432, df = 1, p-value = 0.001705
```

Create PDF in your applications with the Pdfcrowd HTML to PDF API PDFCROWD

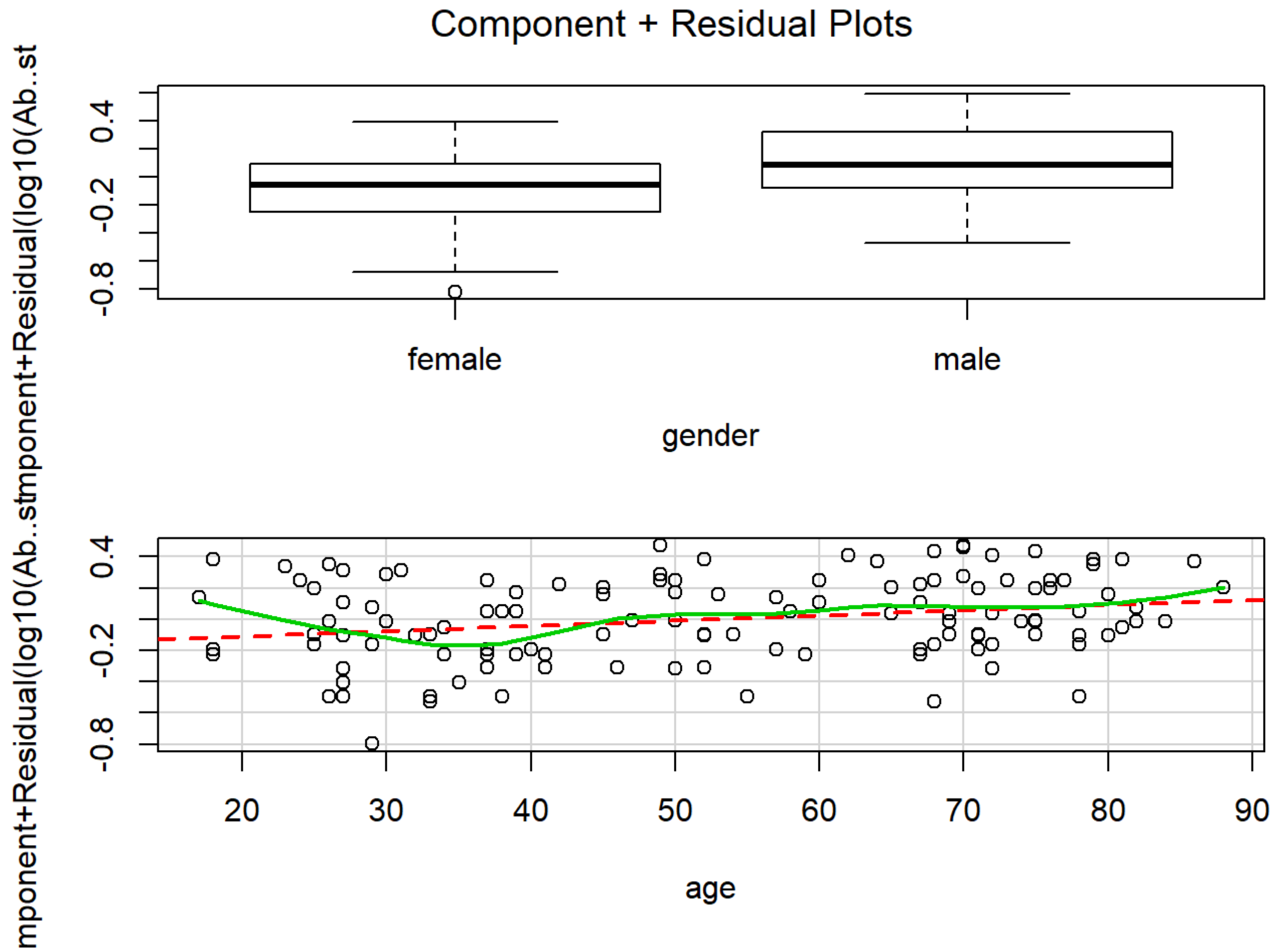

Figure 19. Component+residual plot to check linearity.

Create PDF in your applications with the Pdfcrowd HTML to PDF API PDFCROWD

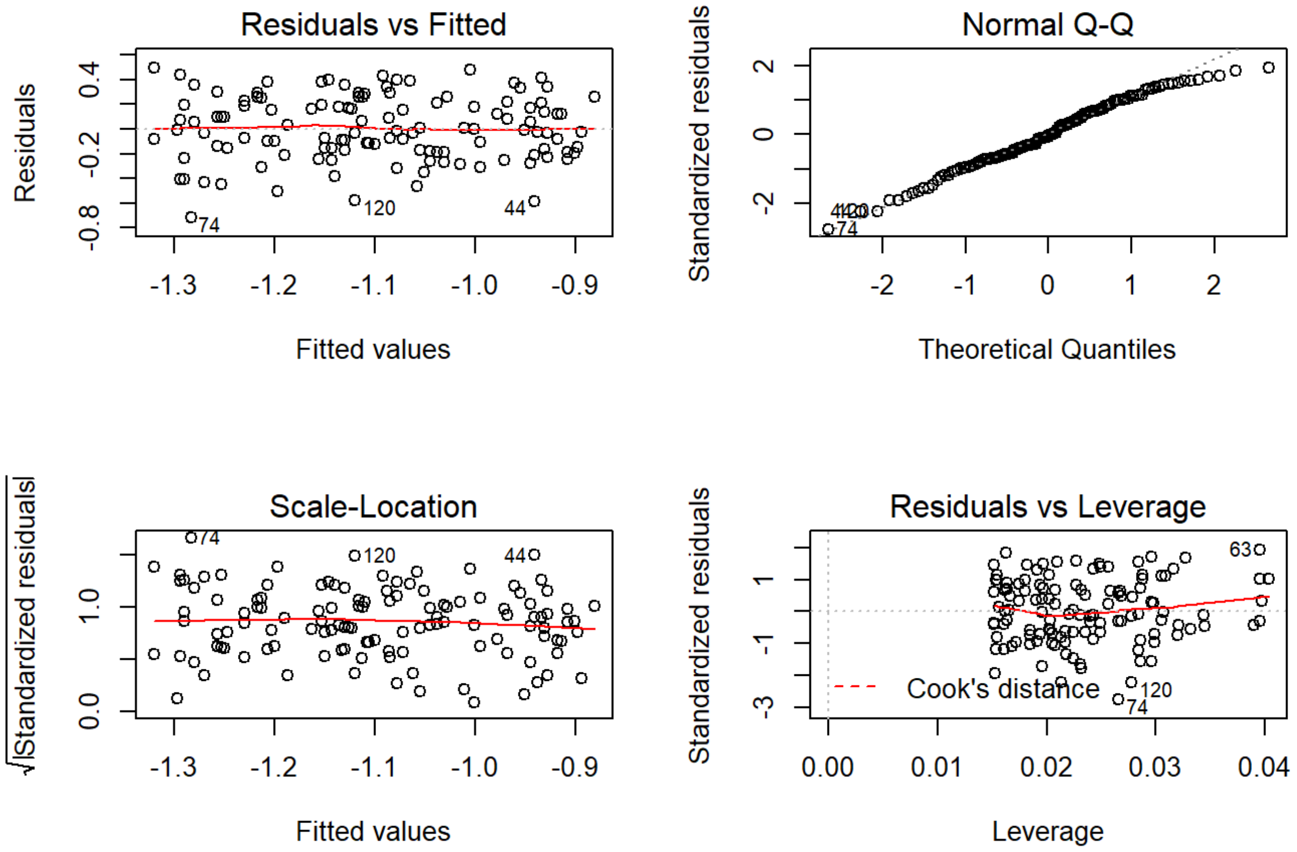

Figure 20. Plots to assess assumptions of homoscedasticity and normality.

```
##
##  studentized Breusch-Pagan test
##
## data:  mod5b
## BP = 2.5883, df = 1, p-value = 0.1077
```

```
## Analysis of Variance Table
##
```

Create PDF in your applications with the Pdfcrowd HTML to PDF API PDFCROWD

```
## Response: log10(Ab..stroke.volume)
##            Df Sum Sq Mean Sq F value    Pr(>F)    
## gender      1 1.3852 1.38519 20.1227 1.625e-05 ***
## age         1 0.5589 0.55887  8.1188  0.005125 ** 
## Residuals 125 8.6047 0.06884                      
## ---
## Signif. codes:  0 '***' 0.001 '**' 0.01 '*' 0.05 '.' 0.1 ' ' 1
```

```
## 
## Call:
## lm(formula = log10(Ab..stroke.volume) ~ gender + age, data = mydata)
## 
## Residuals:
##     Min      1Q  Median      3Q     Max 
## -0.7161 -0.1783 -0.0126  0.1996  0.4967 
## 
## Coefficients:
##              Estimate Std. Error t value Pr(>|t|)    
## (Intercept) -1.380786   0.069973 -19.733  < 2e-16 ***
## gendermale   0.211893   0.046422   4.565 1.18e-05 ***
## age          0.003342   0.001173   2.849  0.00513 ** 
## ---
## Signif. codes:  0 '***' 0.001 '**' 0.01 '*' 0.05 '.' 0.1 ' ' 1
## 
## Residual standard error: 0.2624 on 125 degrees of freedom
## Multiple R-squared:  0.1843, Adjusted R-squared:  0.1712 
## F-statistic: 14.12 on 2 and 125 DF,  p-value: 2.957e-06
```

```
##                   2.5 %       97.5 %
## (Intercept) -1.519271653 -1.242299773
## gendermale   0.120018525  0.303767690
## age          0.001020678  0.005663304
```

Mean flux

Create PDF in your applications with the Pdfcrowd HTML to PDF API PDFCROWD

An ordinal regression was calculated for Mean.flux that was recoded into a variable with four categories. The test of nominal effects indicated that the proportional odds assumption was met. Furthermore, the distances between the symbols for each set of categories of the dependent variable remained fairly similar (Fig. 21). The first set of coefficients was set to zero to have a common reference point.
The ordinal regression indicates that both gender ($p<0.001$) and age ($p<0.001$) are significant predictors for Mean.flux. As age increases by one unit, the odds of observing category 4 of Mean.flux vs. the other 3 categories increase by a factor of 1.03. The odds of observing category 4 of Mean.flux vs. the other 3 categories are 4.51 times higher for male than for female probands.

```
## Call:
## polr(formula = Mean.flux.rec ~ gender + age, data = mydata, Hess = TRUE)
##
## Coefficients:
##              Value Std. Error t value
## gendermale 1.50672   0.357267   4.217
## age        0.03116   0.008982   3.469
##
## Intercepts:
##     Value   Std. Error t value
## 1|2 -0.4717  0.5502    -0.8573
## 2|3  2.4569  0.5590     4.3951
## 3|4  3.7905  0.6223     6.0908
##
## Residual Deviance: 287.8905
## AIC: 297.8905
```

```
##                  Value  Std. Error    t value      p value
## gendermale  1.50672101 0.357266853  4.2173546 2.471851e-05
## age         0.03116203 0.008981721  3.4694938 5.214402e-04
## 1|2        -0.47169415 0.550191837 -0.8573267 3.912644e-01
## 2|3         2.45688797 0.559008932  4.3950782 1.107329e-05
## 3|4         3.79047337 0.622328426  6.0907926 1.123530e-09
```

```
##                  OR    2.5 %   97.5 %
## gendermale 4.511912 2.269267 9.239867
## age        1.031653 1.013945 1.050376
```

Create PDF in your applications with the Pdfcrowd HTML to PDF API PDFCROWD

```
## as.numeric(Mean.flux.rec)    N=128
##
## +-------+-------+---+----+----+---------+---------+
## |       |       |N  |Y>=1|Y>=2|Y>=3     |Y>=4     |
## +-------+-------+---+----+----+---------+---------+
## |gender |female | 66|Inf |0   |-2.743142|-4.112629|
## |       |male   | 62|Inf |0   |-2.873130|-3.999034|
## +-------+-------+---+----+----+---------+---------+
## |age    |[17,35)| 32|Inf |0   |-2.779509|-4.214594|
## |       |[35,53)| 35|Inf |0   |-3.453948|-5.170484|
## |       |[53,72)| 32|Inf |0   |-2.143520|-3.057141|
## |       |[72,88]| 29|Inf |0   |-1.960836|-2.950996|
## +-------+-------+---+----+----+---------+---------+
## |Overall|       |128|Inf |0   |-2.593263|-3.695924|
## +-------+-------+---+----+----+---------+---------+
```

Create PDF in your applications with the Pdfcrowd HTML to PDF API PDFCROWD

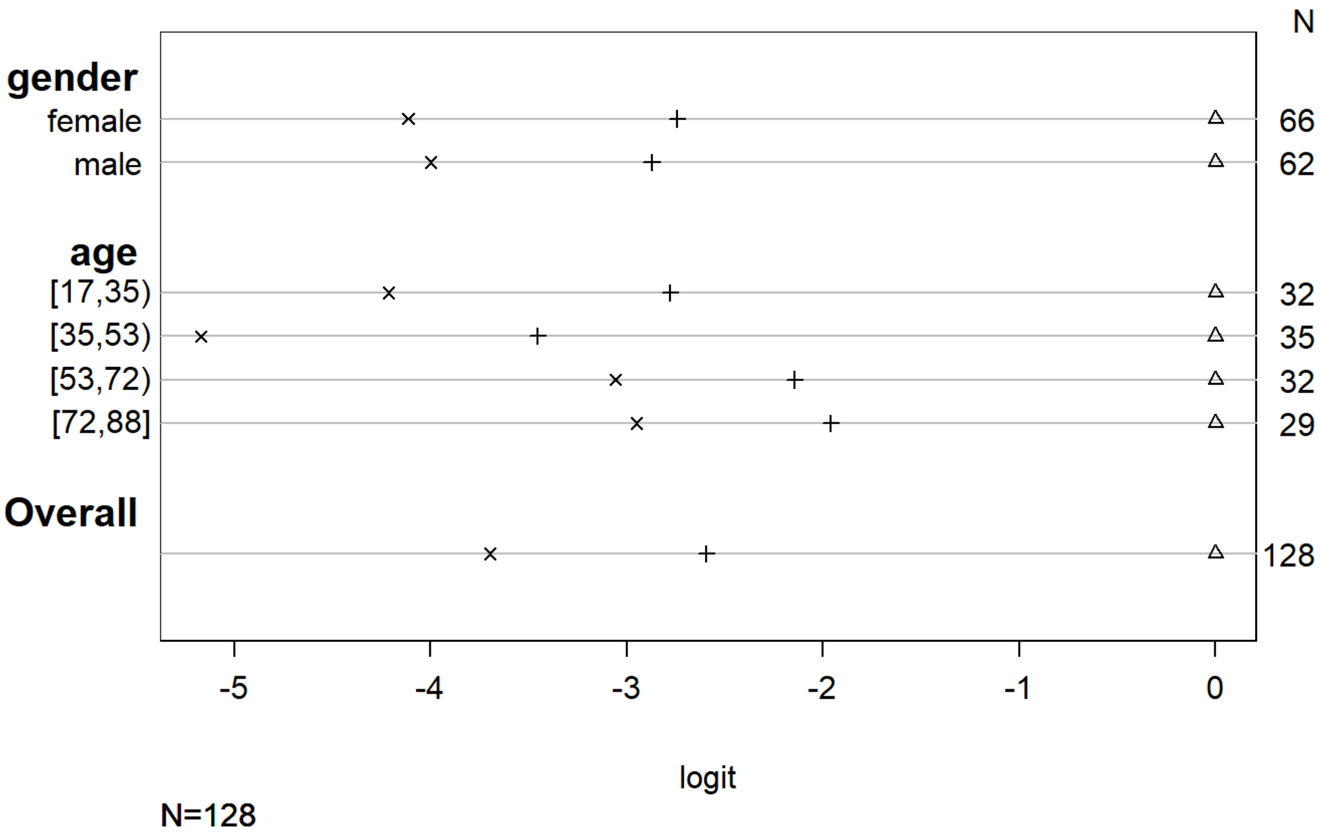

Figure 21. Checking proportional odds assumption. The x, plus, and triangle sign correspond to Y >=4,3,2, respectively.

```
## Tests of nominal effects
##
## formula: Mean.flux.rec ~ gender + age
##         Df  logLik    AIC    LRT Pr(>Chi)
## <none>     -143.94 297.89
## gender  2 -143.83 301.66 0.2260   0.8931
## age     2 -141.68 297.36 4.5305   0.1038
```

Create PDF in your applications with the Pdfcrowd HTML to PDF API PDFCROWD

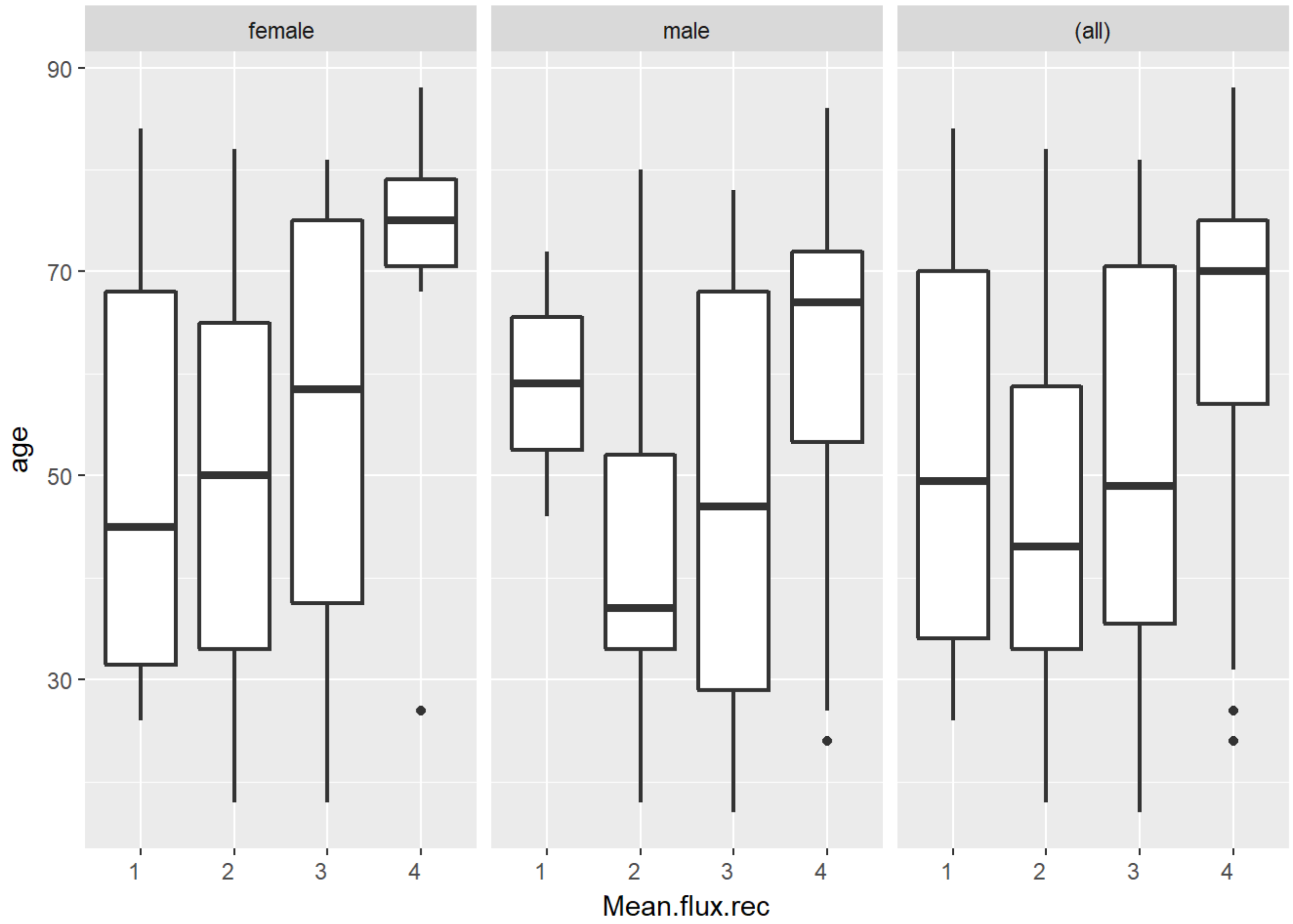

Figure 22. Boxplots for age for every level of Mean.flux and gender.

stroke distance

Model mod7a (stroke.distance ~ gender + age) did not meet assumption of homoscedasticity (see studentized Breusch-Pagan test). Therefore, stroke.distance was logarithmized. Model mod7b met all assumptions. Thus, age was fairly linearly related to stroke.distance (Fig. 23), assumptions of homoscedasticity (Fig. 24, residuals vs fitted values plot, scale-location plot, and Breusch-Pagan test) and normality (Fig. 24, QQ plot) were also met.
The analysis of variance table indicates that neither gender (p=0.083) nor age (p=0.114) are significant predictors for stroke.distance.

Create PDF in your applications with the Pdfcrowd HTML to PDF API PDFCROWD

```
##
##  studentized Breusch-Pagan test
##
## data:  mod7a
## BP = 4.9276, df = 1, p-value = 0.02643
```

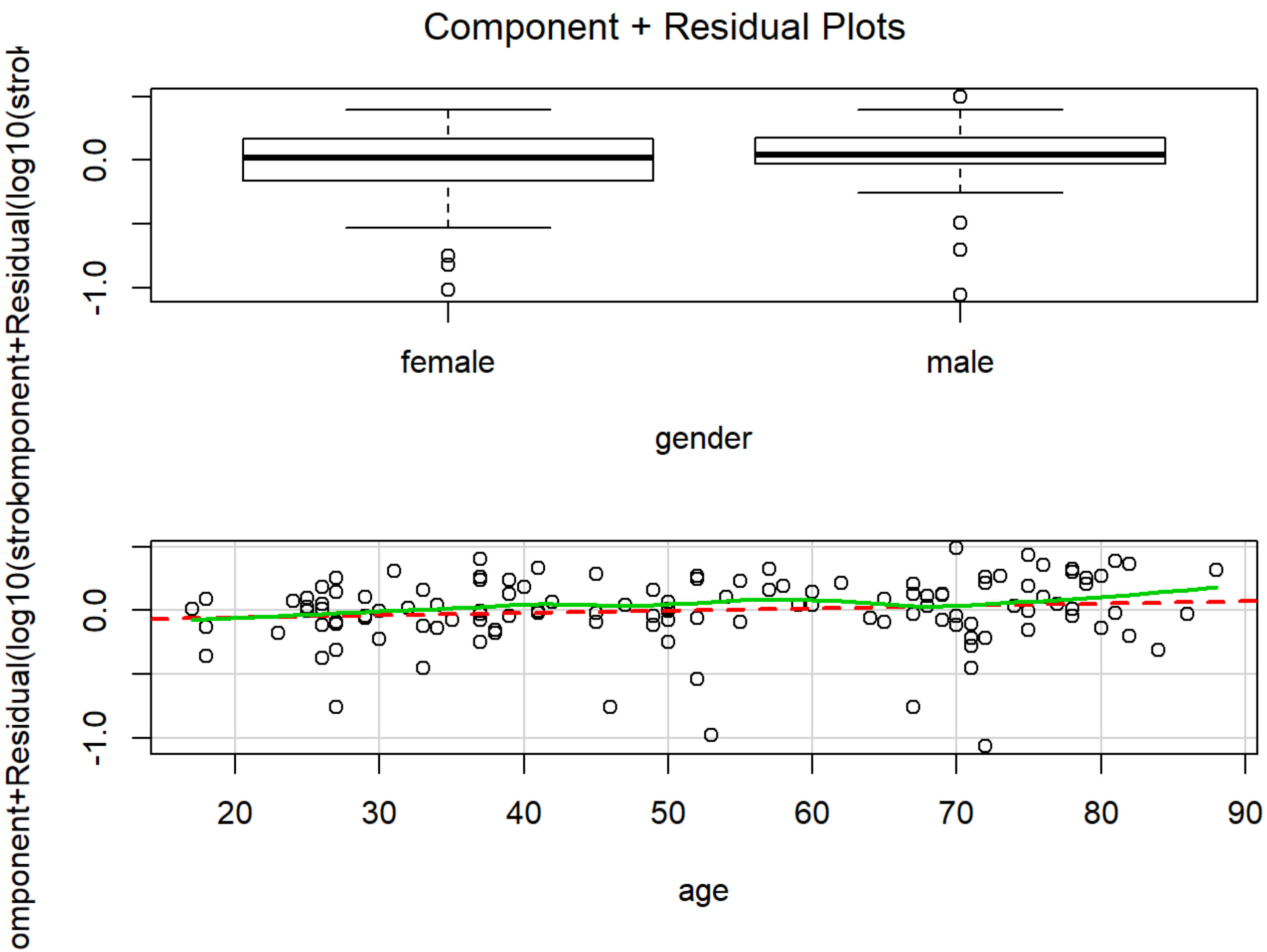

Figure 23. Component+residual plot to check linearity.

Create PDF in your applications with the Pdfcrowd HTML to PDF API PDFCROWD

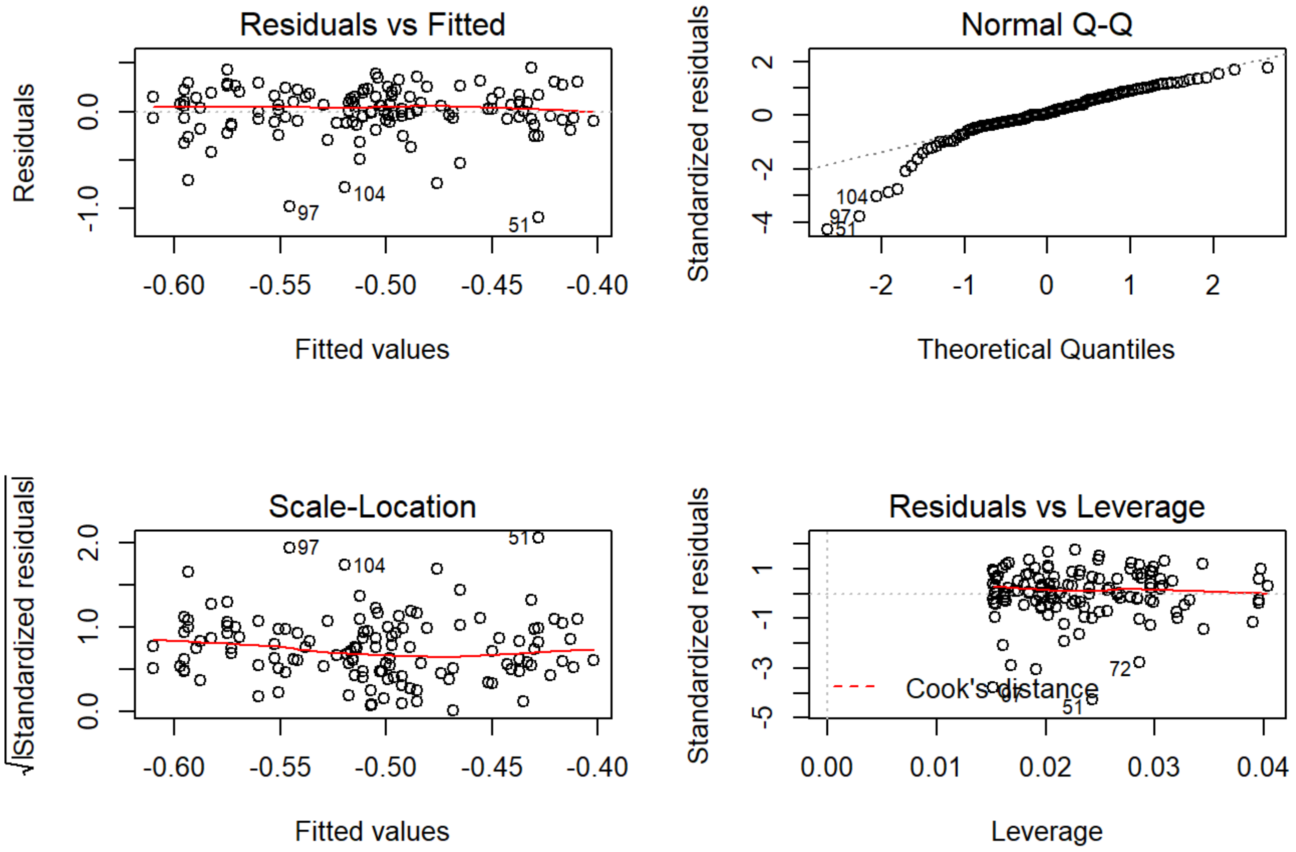

Figure 24. Plots to assess assumptions of homoscedasticity and normality.

```
##
##  studentized Breusch-Pagan test
##
## data:  mod7b
## BP = 0.015376, df = 1, p-value = 0.9013
```

```
## Analysis of Variance Table
##
```

Create PDF in your applications with the Pdfcrowd HTML to PDF API PDFCROWD

```
## Response: log10(stroke.distance)
##            Df Sum Sq  Mean Sq F value  Pr(>F)
## gender      1 0.2073 0.207263  3.0607 0.08266 .
## age         1 0.1715 0.171457  2.5319 0.11409
## Residuals 125 8.4647 0.067718
## ---
## Signif. codes:  0 '***' 0.001 '**' 0.01 '*' 0.05 '.' 0.1 ' ' 1
```

```
##
## Call:
## lm(formula = log10(stroke.distance) ~ gender + age, data = mydata)
##
## Residuals:
##      Min       1Q   Median       3Q      Max
## -1.09512 -0.09224  0.01505  0.16827  0.45265
##
## Coefficients:
##              Estimate Std. Error t value Pr(>|t|)
## (Intercept) -0.643626   0.069402  -9.274 6.78e-16 ***
## gendermale   0.082588   0.046043   1.794   0.0753 .
## age          0.001851   0.001163   1.591   0.1141
## ---
## Signif. codes:  0 '***' 0.001 '**' 0.01 '*' 0.05 '.' 0.1 ' ' 1
##
## Residual standard error: 0.2602 on 125 degrees of freedom
## Multiple R-squared:  0.04282,    Adjusted R-squared:  0.02751
## F-statistic: 2.796 on 2 and 125 DF,  p-value: 0.06486
```

```
##                     2.5 %      97.5 %
## (Intercept) -0.7809811206 -0.506270267
## gendermale  -0.0085364303  0.173712720
## age         -0.0004512798  0.004153446
```

Mean velocity

Create PDF in your applications with the Pdfcrowd HTML to PDF API PDFCROWD

Model mod8a (Mean.velocity ~ gender + age) did not meet assumption of homoscedasticity (see studentized Breusch-Pagan test). Therefore, Mean.velocity was logarithmized. Model mod8b met all assumptions. Thus, age was fairly linearly related to Mean.velocity (Fig. 25), assumptions of homoscedasticity (Fig. 26, residuals vs fitted values plot, scale-location plot, and Breusch-Pagan test) and normality (Fig. 26, QQ plot) were also met.
The analysis of variance table indicates that neither gender(p=0.141) nor age (p=0.084) are significant predictors for Mean.velocity.

```
##
##  studentized Breusch-Pagan test
##
## data:  mod8a
## BP = 6.236, df = 1, p-value = 0.01252
```

Create PDF in your applications with the Pdfcrowd HTML to PDF API PDFCROWD

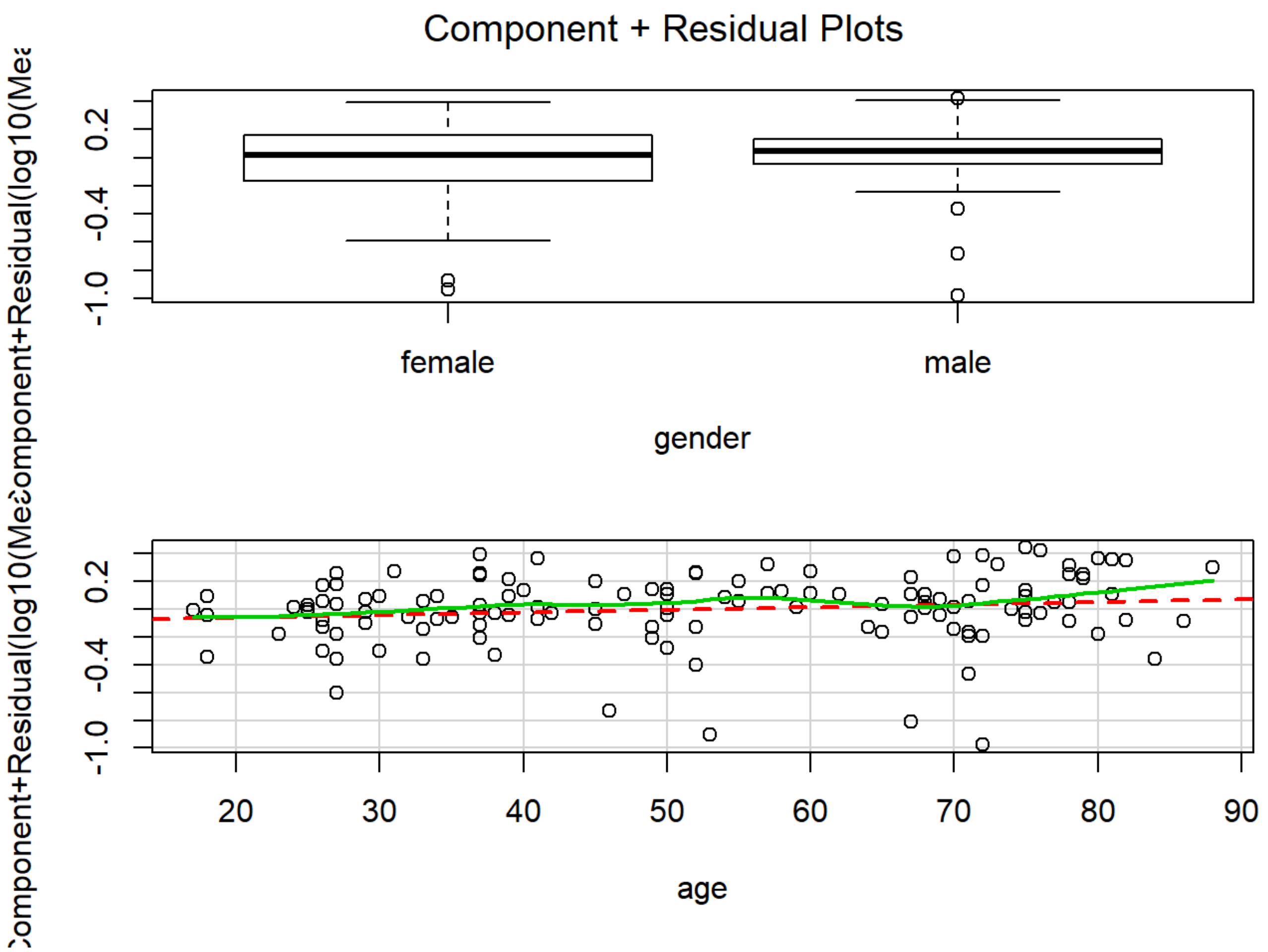

Figure 25. Component+residual plot to check linearity.

Create PDF in your applications with the Pdfcrowd HTML to PDF API PDFCROWD

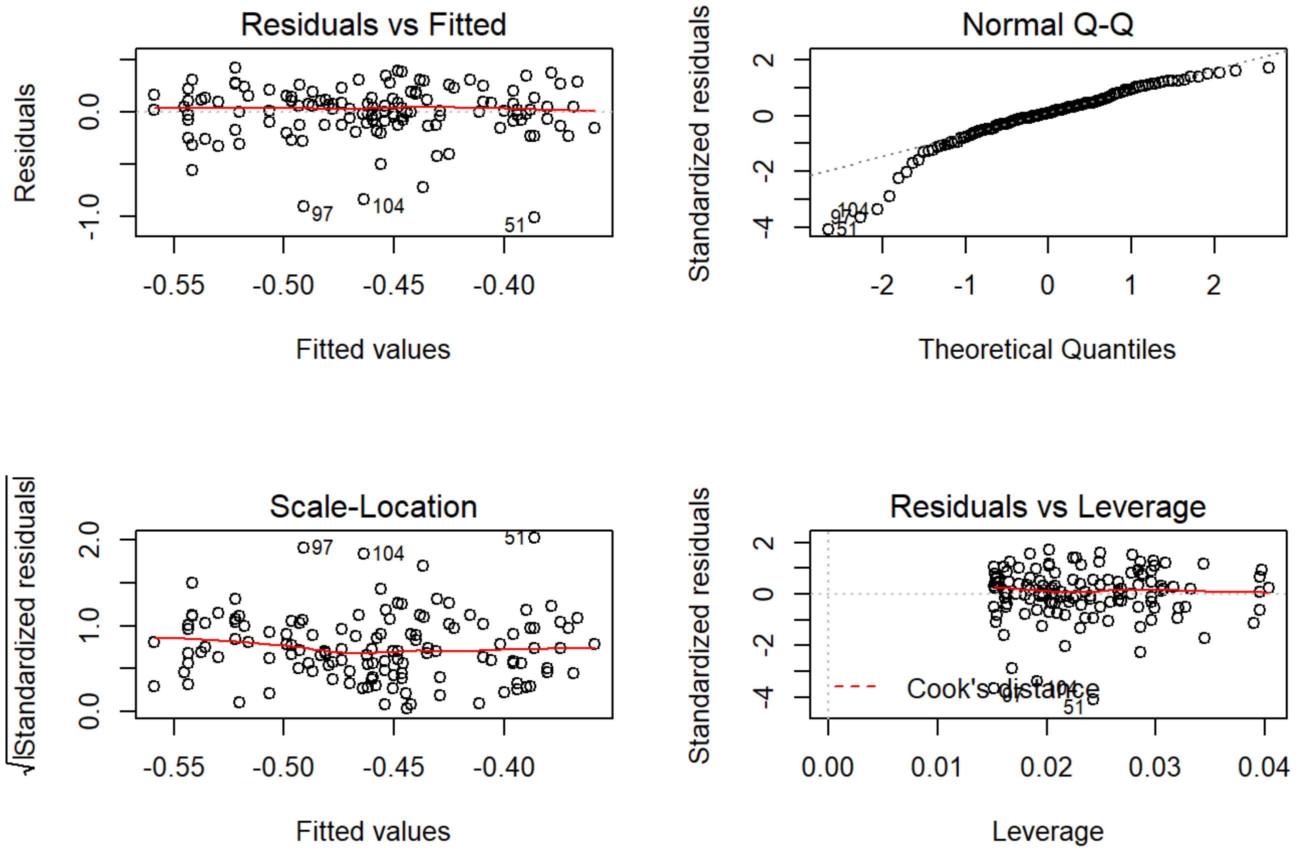

Figure 26. Plots to assess assumptions of homoscedasticity and normality.

```
##
##  studentized Breusch-Pagan test
##
## data:  mod8b
## BP = 0.12285, df = 1, p-value = 0.726
```

```
## Analysis of Variance Table
##
```

Create PDF in your applications with the Pdfcrowd HTML to PDF API PDFCROWD

```
## Response: log10(Mean.velocity)
##            Df Sum Sq  Mean Sq F value  Pr(>F)
## gender      1 0.1376 0.137590  2.1939 0.14108
## age         1 0.1903 0.190331  3.0348 0.08396 .
## Residuals 125 7.8395 0.062716
## ---
## Signif. codes:  0 '***' 0.001 '**' 0.01 '*' 0.05 '.' 0.1 ' ' 1
```

```
##
## Call:
## lm(formula = log10(Mean.velocity) ~ gender + age, data = mydata)
##
## Residuals:
##      Min       1Q   Median       3Q      Max
## -1.01191 -0.11002  0.02149  0.15042  0.42517
##
## Coefficients:
##             Estimate Std. Error t value Pr(>|t|)
## (Intercept) -0.59424    0.06679  -8.897 5.46e-15 ***
## gendermale   0.06778    0.04431   1.530    0.129
## age          0.00195    0.00112   1.742    0.084 .
## ---
## Signif. codes:  0 '***' 0.001 '**' 0.01 '*' 0.05 '.' 0.1 ' ' 1
##
## Residual standard error: 0.2504 on 125 degrees of freedom
## Multiple R-squared:  0.04015,    Adjusted R-squared:  0.02479
## F-statistic: 2.614 on 2 and 125 DF,  p-value: 0.07722
```

```
##                     2.5 %       97.5 %
## (Intercept) -0.7264237205 -0.462053178
## gendermale  -0.0199105250  0.155478639
## age         -0.0002653923  0.004166009
```

Peak velocity

Create PDF in your applications with the Pdfcrowd HTML to PDF API PDFCROWD

Model mod8a (Mean.velocity ~ gender + age) met all assumptions. Thus, age was fairly linearly related to Mean.velocity (Fig. 27), assumptions of homoscedasticity (Fig. 28, residuals vs fitted values plot, scale-location plot, and Breusch-Pagan test) and normality (Fig. 28, QQ plot) were also met. The analysis of variance table indicates that both gender (p=0.009) and age (p=0.016) were a significant predictors for Mean.velocity.

```
##
##  studentized Breusch-Pagan test
##
## data:  mod9a
## BP = 1.1301, df = 1, p-value = 0.2877
```

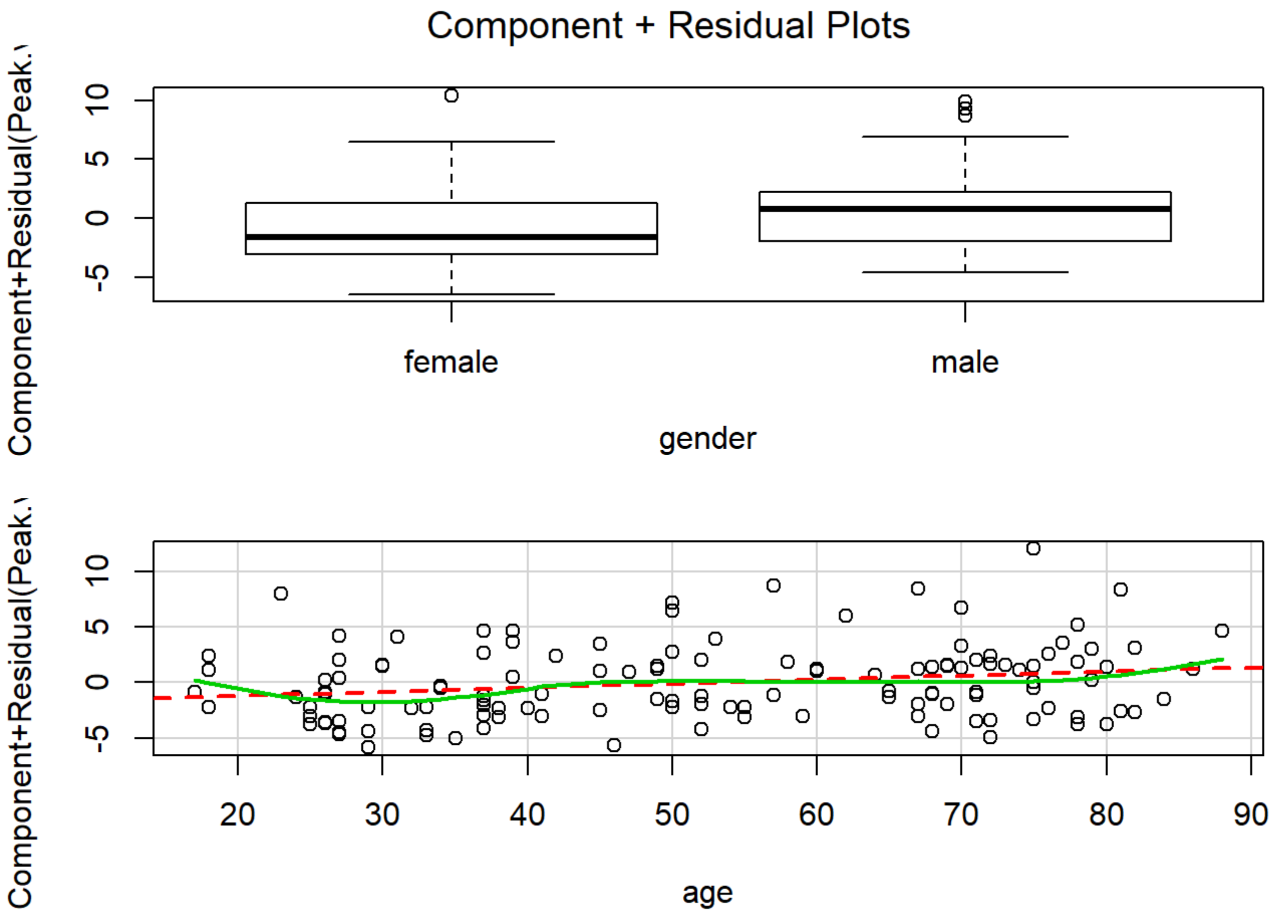

Create PDF in your applications with the Pdfcrowd HTML to PDF API PDFCROWD

Figure 27. Component+residual plot to check linearity.

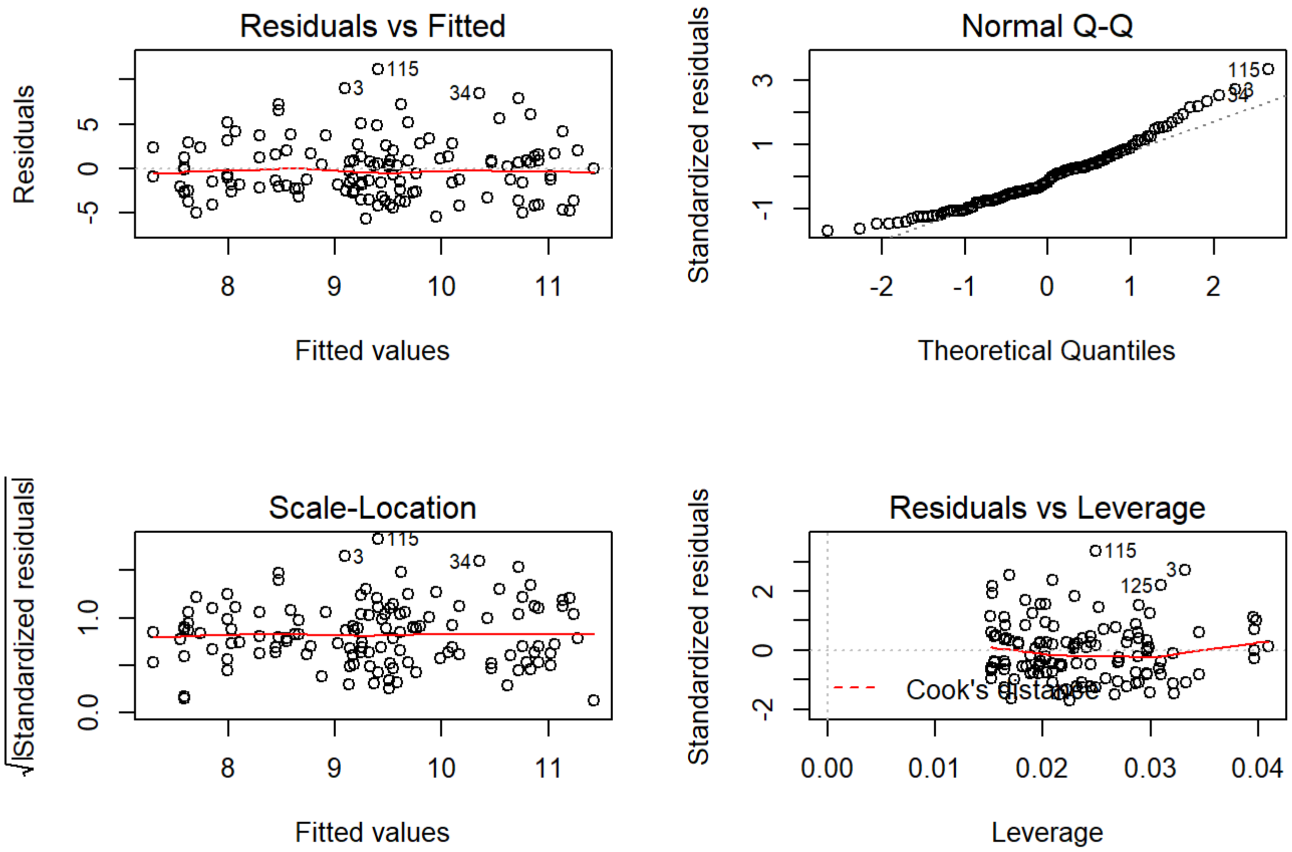

Figure 28. Plots to assess assumptions of homoscedasticity and normality.

```
## Analysis of Variance Table
##
## Response: Peak.velocity
##            Df  Sum Sq Mean Sq F value   Pr(>F)
## gender      1   79.44  79.439  6.9815 0.009297 **
## age         1   68.15  68.153  5.9896 0.015791 *
## Residuals 124 1410.93  11.378
```

Create PDF in your applications with the Pdfcrowd HTML to PDF API PDFCROWD

```
## ---
## Signif. codes:  0 '***' 0.001 '**' 0.01 '*' 0.05 '.' 0.1 ' ' 1
```

```
##
## Call:
## lm(formula = Peak.velocity ~ gender + age, data = mydata)
##
## Residuals:
##     Min      1Q  Median      3Q     Max
## -5.6715 -2.5128 -0.6138  1.6844 11.1777
##
## Coefficients:
##             Estimate Std. Error t value Pr(>|t|)
## (Intercept)  6.63115    0.90047   7.364 2.17e-11 ***
## gendermale   1.61776    0.59928   2.700  0.00791 **
## age          0.03695    0.01510   2.447  0.01579 *
## ---
## Signif. codes:  0 '***' 0.001 '**' 0.01 '*' 0.05 '.' 0.1 ' ' 1
##
## Residual standard error: 3.373 on 124 degrees of freedom
##   (1 observation deleted due to missingness)
## Multiple R-squared:  0.0947, Adjusted R-squared:  0.0801
## F-statistic: 6.486 on 2 and 124 DF,  p-value: 0.002095
```

```
##                   2.5 %     97.5 %
## (Intercept) 4.848873834 8.41343515
## gendermale  0.431620190 2.80390236
## age         0.007066984 0.06683156
```

Peak pressure gradient

Model mod10a (Peak.pressure.gradient ~ gender + age) met all assumptions. Thus, age was fairly linearly related to Peak.pressure.gradient (Fig. 29), assumptions of homoscedasticity (Fig. 30, residuals vs fitted values plot, scale-location plot, and Breusch-Pagan test) and normality (Fig. 30,

Create PDF in your applications with the Pdfcrowd HTML to PDF API PDFCROWD

QQ plot) were also met.

The analysis of variance table indicates that both gender (p=0.029) and age (p=0.028) are significant predictors for Peak.pressure.gradient.

```
##
##  studentized Breusch-Pagan test
##
## data:  mod10a
## BP = 1.9856, df = 1, p-value = 0.1588
```

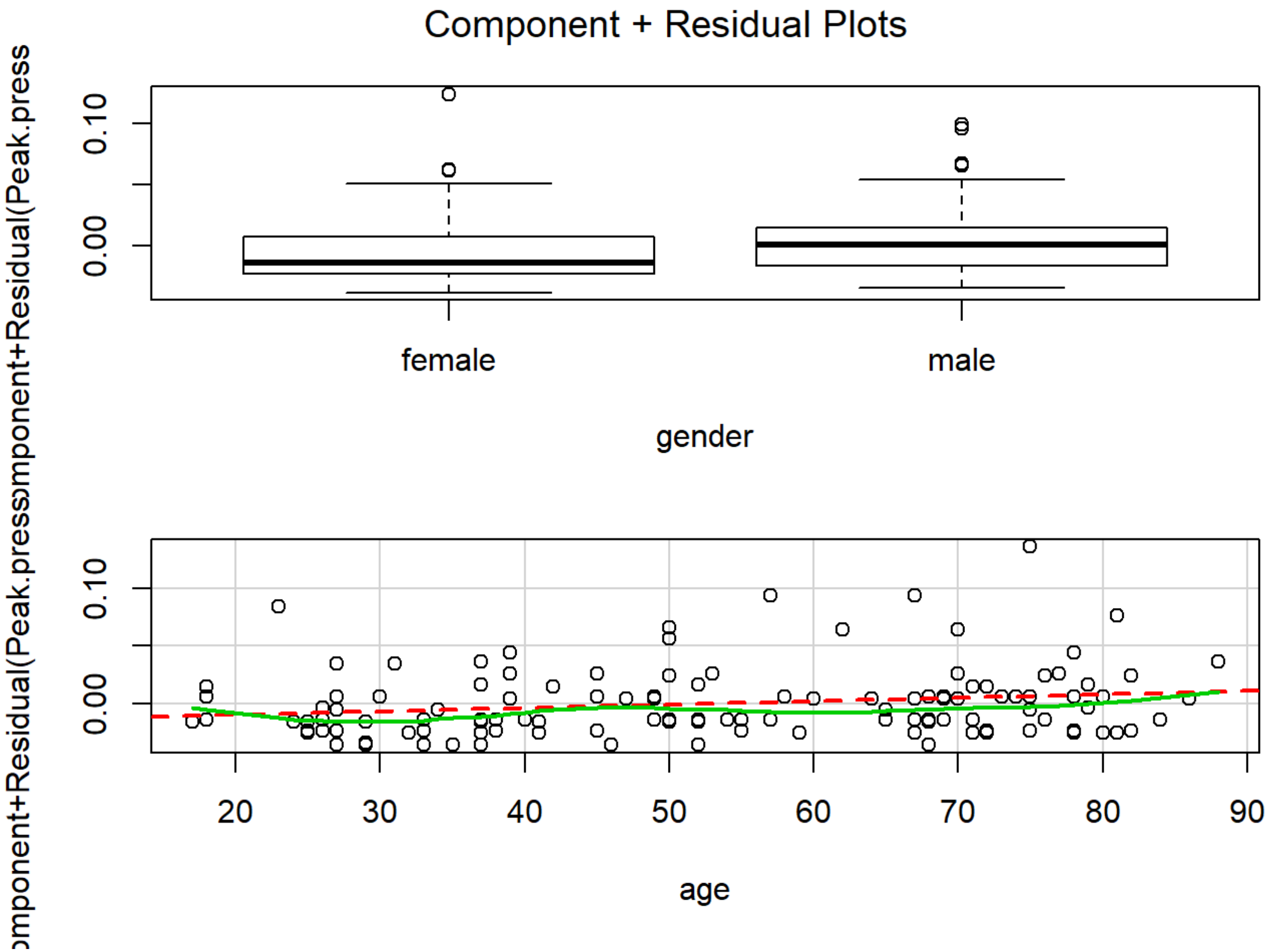

Figure 29. Component+residual plot to check linearity.

Create PDF in your applications with the Pdfcrowd HTML to PDF API PDFCROWD

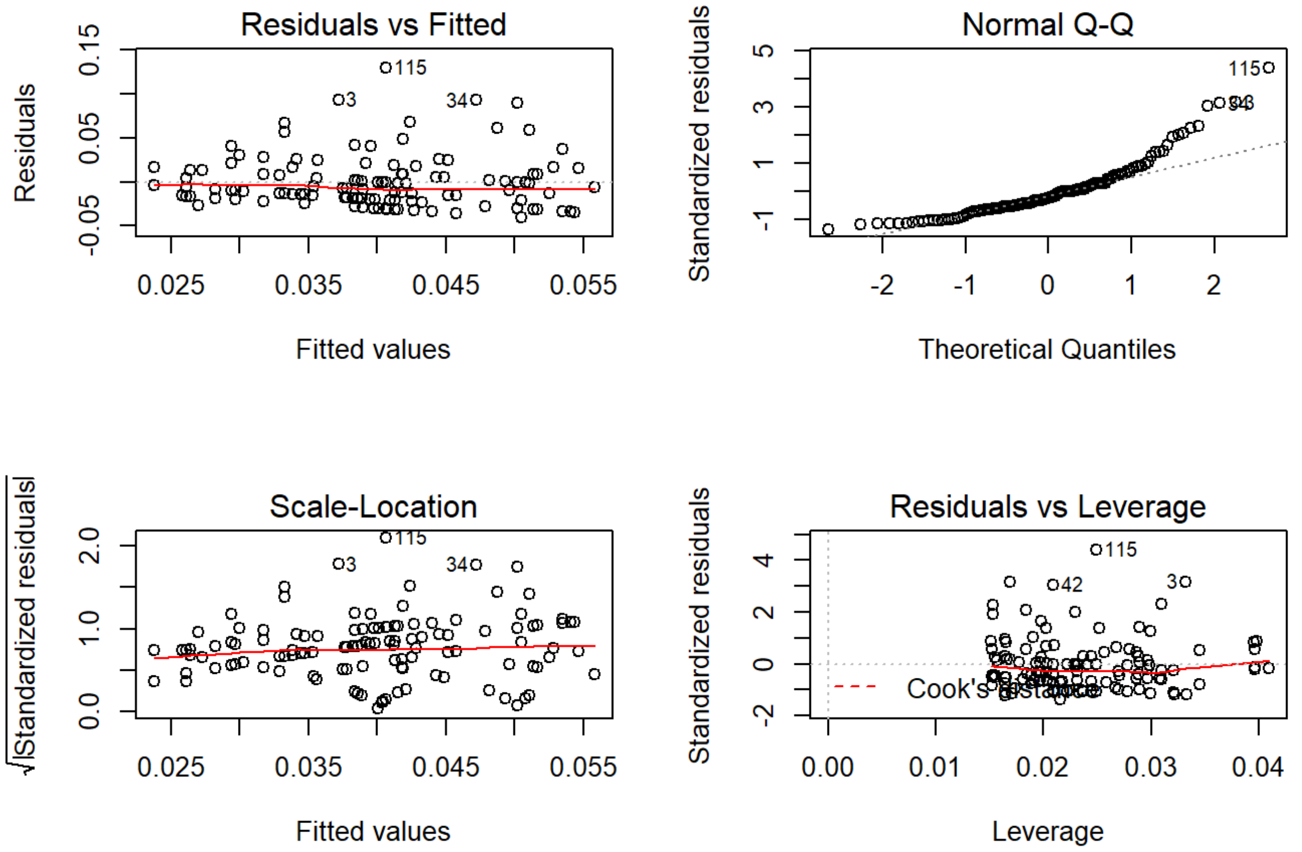

Figure 30. Plots to assess assumptions of homoscedasticity and normality.

```
## Analysis of Variance Table
##
## Response: Peak.pressure.gradient
##            Df   Sum Sq   Mean Sq F value  Pr(>F)
## gender      1 0.004300 0.0043001  4.8510 0.02948 *
## age         1 0.004368 0.0043678  4.9273 0.02825 *
## Residuals 124 0.109919 0.0008864
```

Create PDF in your applications with the Pdfcrowd HTML to PDF API PDFCROWD

```
## ---
## Signif. codes:  0 '***' 0.001 '**' 0.01 '*' 0.05 '.' 0.1 ' ' 1
```

```
##
## Call:
## lm(formula = Peak.pressure.gradient ~ gender + age, data = mydata)
##
## Residuals:
##       Min        1Q    Median        3Q       Max
## -0.040475 -0.018698 -0.007460  0.008489  0.129379
##
## Coefficients:
##              Estimate Std. Error t value Pr(>|t|)
## (Intercept) 0.0184359  0.0079479   2.320   0.0220 *
## gendermale  0.0119250  0.0052895   2.254   0.0259 *
## age         0.0002958  0.0001333   2.220   0.0283 *
## ---
## Signif. codes:  0 '***' 0.001 '**' 0.01 '*' 0.05 '.' 0.1 ' ' 1
##
## Residual standard error: 0.02977 on 124 degrees of freedom
##   (1 observation deleted due to missingness)
## Multiple R-squared:  0.07309,    Adjusted R-squared:  0.05814
## F-statistic: 4.889 on 2 and 124 DF,  p-value: 0.009041
```

```
##                    2.5 %       97.5 %
## (Intercept) 2.704801e-03 0.0341670919
## gendermale  1.455664e-03 0.0223944066
## age         3.204606e-05 0.0005595529
```

References

R Core Team (2016). R: A language and environment for statistical computing. R Foundation for Statistical Computing, Vienna, Austria. URL https://www.R-project.org/.

Venables, W. N. & Ripley, B. D. (2002) Modern Applied Statistics with S. Fourth Edition. Springer, New York. ISBN 0-387-95457-0

Create PDF in your applications with the Pdfcrowd HTML to PDF API PDFCROWD

Create PDF in your applications with the Pdfcrowd HTML to PDF API PDFCROWD
